# Branched‐Chain α Keto‐Acid Dehydrogenase Kinase‐Mediated AKT Phosphorylation Promotes RCC Tumorigenesis and Drug Resistance

**DOI:** 10.1002/advs.202411081

**Published:** 2025-08-11

**Authors:** Qin Tian, Jinxiang Wang, Qiji Li, Yangruiyu Liu, Yanping Chen, Jiacheng Feng, Zi‐Ning Lei, Harsh Patel, Chao‐Yun Cai, Yuzhi Xu, Chuntao Quan, Lingyan Fei, Zexiu Xiao, Shuo Fang, Tianxin Lin, Zhe‐Sheng Chen, Yuchen Liu, Leli Zeng, Yihang Pan

**Affiliations:** ^1^ Guangdong Provincial Key Laboratory of Digestive Cancer Research Precision Medicine Center The Biobank Scientific Research Center The Seventh Affiliated Hospital School of Medicine Sun Yat‐Sen University Shenzhen Guangdong 518107 China; ^2^ Department of Orthopaedic Surgery The Seventh Affiliated Hospital Sun Yat‐sen University Shenzhen Guangdong 518107 China; ^3^ The Pharmacy College of Jiangxi Science and Technology Normal University Nanchang Jiangxi 330013 China; ^4^ Department of Pharmaceutical Sciences College of Pharmacy and Health Sciences St. John's University Queens NY 11439 USA; ^5^ Biobank Shenzhen Second People's Hospital Shenzhen Guangdong 518035 China; ^6^ Department of Nephrology Center of Kidney and Urology the Seventh Affiliated Hospital Sun Yat‐sen University Shenzhen Guangdong 518107 China; ^7^ Shenzhen MagicRNA Biotech Shenzhen Guangdong China; ^8^ Department of Oncology The Seventh Affiliated Hospital Sun Yat‐sen University Shenzhen Guangdong 518107 China; ^9^ Guangdong Provincial Key Laboratory of Malignant Tumor Epigenetics and Gene Regulation Department of Urology Sun Yat‐sen Memorial Hospital Sun Yat‐sen University Guangzhou Guangdong 510120 China

**Keywords:** AKT phosphorylation, apoptosis, branched‐chain α keto‐acid dehydrogenase kinase, drug resistance, PDOs

## Abstract

Advanced renal cell carcinoma (RCC) primarily relies on targeted and immune‐based therapies, yet these treatments often face limitations due to inefficacy and drug resistance. Branched‐chain α‐keto‐acid dehydrogenase kinase (BCKDK) has been implicated in promoting RCC metastasis, but its specific substrates and the mechanisms underlying its regulation of RCC progression remain poorly understood. This study uncovers a novel mechanism whereby BCKDK‐mediated AKT phosphorylation drives RCC tumorigenesis and drug resistance. Elevated BCKDK expression correlates with poor prognosis in RCC clinical samples. BCKDK deficiency inhibits RCC cell proliferation and tumorigenesis both in vitro and in vivo. Mechanistic investigations reveal that BCKDK directly binds to and regulates the phosphorylation of AKT. BCKDK‐mediated phosphorylation of AKT decreases ubiquitin‐mediated AKT protein degradation, and promotes tumorigenesis via activation of the AKT/mTOR signaling pathway. RNA sequencing identifies BCKDK's involvement in the drug metabolism network and apoptotic signaling pathways. The BCKDK/AKT/ABCB1 axis mediates doxorubicin resistance. Targeting BCKDK/AKT inhibits the growth of RCC patient‐derived organoids (PDOs), enhances doxorubicin‐induced apoptosis in RCC cells, and suppresses tumor growth in vivo. These findings identify a previously unrecognized phosphorylation substrate of BCKDK and highlight the critical role of the BCKDK/AKT signaling axis in RCC progression, offering a promising target for therapeutic intervention.

## Graphical abstract

This study identifies a novel oncogenic role and a previously unrecognized phosphorylation substrate of BCKDK in RCC, wherein it promotes tumor progression and drug resistance through AKT phosphorylation at both Thr308 and Ser473 sites and activation of AKT/mTOR and AKT/ABCB1 signaling pathways, offering a promising prognostic marker and therapeutic target for RCC.

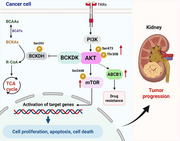

## 1 Introduction

Kidney cancer ranks among the most prevalent malignancies of the urinary system, with an estimated 81610 new cases and 14390 deaths in 2024.^[^ ^1^ ^]^ Histologically, renal cell carcinoma (RCC) constitutes ≈90% of all kidney cancer cases.^[^ ^2^ ^]^ The majority of RCC exhibits resistance to both radiotherapy and chemotherapy, resulting in a poor prognosis for patients with advanced stages.^[^ ^3^, ^4^, ^5^, ^6^ ^]^ The primary cause of chemotherapy failure in RCC is multidrug resistance (MDR), notably to vinblastine, doxorubicin (DOX), and other chemotherapeutic agents. Enhancing the sensitivity of RCC to chemotherapy is critical for improving treatment outcomes and patient survival.^[^ ^7^, ^8^ ^]^ Consequently, understanding the molecular mechanisms driving RCC pathogenesis and identifying novel therapeutic strategies remain urgent priorities.

Branched‐chain amino acids (BCAAs), which include leucine, isoleucine, and valine, are essential for tumor cell growth, survival, proliferation, invasion, and migration.^[^ ^9^, ^10^ ^]^ The pivotal role of BCAAs metabolic reprogramming has been recognized in various cancers, including leukemia,^[^ ^11^, ^12^ ^]^ pancreatic ductal adenocarcinoma (PDAC),^[^ ^13^, ^14^ ^]^ non‐small cell lung cancer (NSCLC),^[^ ^15^ ^]^ gastric cancer,^[^ ^16^ ^]^ RCC,^[^ ^17^ ^]^ glioblastoma multiforme (GBM),^[^ ^18^ ^]^ and hepatocellular carcinoma (HCC).^[^ ^19^ ^]^ Mechanistic studies have elucidated the contribution of BCAAs metabolism to tumor progression and chemoresistance, suggesting that targeting BCAAs metabolism and related metabolic regulatory enzymes offers a promising therapeutic avenue for cancer treatment.

Branched‐chain α‐keto‐acid dehydrogenase kinase (BCKDK, also known as BDK) plays a pivotal regulatory role in the catabolic pathway of BCAAs by inhibiting the activity of the downstream branched‐chain α‐keto acid dehydrogenase (BCKDH) complex through phosphorylation of the E1‐α subunit at Ser293.^[^ ^20^ ^]^ Previous studies have established a strong association between BCKDK and the progression of various cancers. For instance, aminopeptidase N (APN) regulates the phosphorylation of BCKDK at Ser31, leading to MEK/ERK pathway activation, which promotes proliferation and metastasis in hepatocellular carcinoma cells.^[^ ^21^ ^]^ Our prior research demonstrated that phosphorylation of BCKDK at the Y246 site by upstream Src kinase enhances its activity, contributing to colorectal cancer (CRC) metastasis.^[^ ^22^ ^]^ The therapeutic potential of targeting BCKDK has been highlighted by the efficacy of the BCKDK inhibitor 3,6‐dichlorobenzo1[b]thiophene‐2‐carboxylic acid (BT2), positioning BCKDK as a promising target for cancer and metabolic disease therapy.^[^ ^23^ ^]^ Recent studies also report that the BCKDK/Exosome‐miR‐125a‐5p/VE‐cadherin axis facilitates intercellular communication between RCC cells and human umbilical vein endothelial cells (HUVECs), promoting RCC metastasis.^[^ ^24^ ^]^ However, the precise role and direct substrates of BCKDK in regulating RCC progression remain insufficiently understood.

Our present study indicated that upregulation of BCKDK in clinical RCC samples correlated with poor prognosis. Both in vitro and in vivo data demonstrated that BCKDK promoted tumor proliferation and growth. Mechanistic investigations further revealed that BCKDK interacts with and phosphorylates AKT, thereby activating the AKT/mTOR signaling pathway to drive RCC tumorigenesis. Transcriptomic analysis of BCKDK knockdown RCC cells identified significant alterations in drug metabolism and apoptosis signaling pathways. Specifically, targeting the BCKDK/AKT/P‐glycoprotein (ABCB1) axis enhanced RCC cell sensitivity to DOX‐induced cytotoxicity and apoptosis. Collectively, this study identified a novel downstream substrate of BCKDK and underscored the critical role of BCKDK/AKT signaling in RCC progression, providing a potential strategy for targeted cancer therapy.

## 2 Results

### 2.1 BCKDK is Upregulated in RCC and Predicts a Negative Prognosis

**Figure 1:**
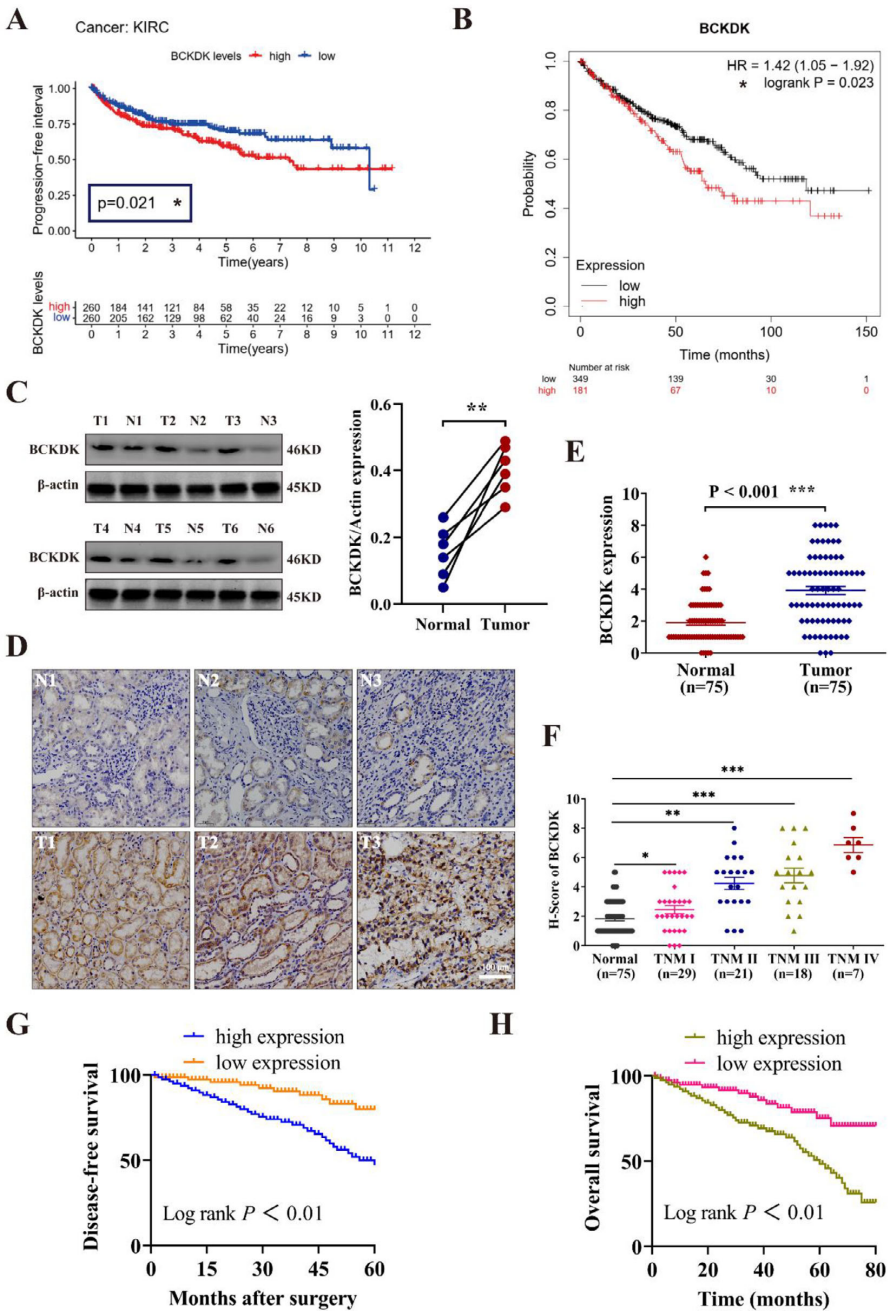
BCKDK is upregulated in RCC and predicts a negative prognosis. A) Correlation between BCKDK expression and disease‐free survival (DFS) in patients with RCC analyzed using the TCGA database via R language. B) Overall survival (OS) analysis of patients with RCC based on BCKDK expression using the Kaplan–Meier Plotter database. C) Western blot analysis of BCKDK protein expression in RCC and normal tissues (n = 6). D) Representative immunohistochemical (IHC) staining images of BCKDK in the tissue microarray (TMA) of clinical RCC samples (75 cases, Cat No. HKidE150CS03, Lot No. XT19‐021). T: tumor; N: normal. E) H‐score of BCKDK expression in RCC TMA (n = 75). F) H‐score of BCKDK expression across different tumor node metastasis (TNM) stages in RCC tissues. G,H) Kaplan–Meier analysis of DFS and OS in patients with RCC grouped by low/high BCKDK expression.

Elevated BCKDK expression has been associated with poor prognosis and metastasis in RCC.^[^ ^24^ ^]^ To validate this, the Cancer Genome Atlas (TCGA) database was first utilized to assess BCKDK expression in RCC. The analysis revealed significant upregulation of BCKDK mRNA in various tumor types, including kidney papillary cell carcinoma (KIRP) and kidney renal clear cell carcinoma (KIRC), compared to their corresponding normal tissues (Figure S1A, Supporting Information). In both paired and unpaired RCC samples, BCKDK mRNA expression was consistently higher in tumor tissues than in normal tissues (Figure S1B,C, Supporting Information). Elevated BCKDK expression was strongly associated with reduced disease‐free survival (DFS) in patients with KIRC (**Figure** **1**A). Kaplan–Meier analysis further confirmed that patients with RCC exhibiting high BCKDK expression experienced significantly poorer overall survival (OS) (Figure 1B). Then, clinical RCC and normal tissue samples were collected, and Western blot analysis confirmed that BCKDK expression was notably higher in tumor tissue compared to normal tissue (Figure 1C). Immunohistochemical (IHC) staining was performed on a tissue microarray (TMA) containing 75 RCC samples and 75 normal samples. The results showed a marked increase in BCKDK protein expression in RCC tissues (Figure 1D,E). High BCKDK expression was positively correlated with advanced tumor node metastasis (TNM) stages in RCC (Figure 1F). In line with TCGA data, patients with higher BCKDK expression exhibited shorter DFS and OS (Figure 1G,H). These results suggested that BCKDK could act as an oncogenic factor in RCC and may serve as a potential prognostic marker for poor clinical outcomes.

### 2.2 BCKDK Promotes RCC Cell Proliferation In Vitro

**Figure 2:**
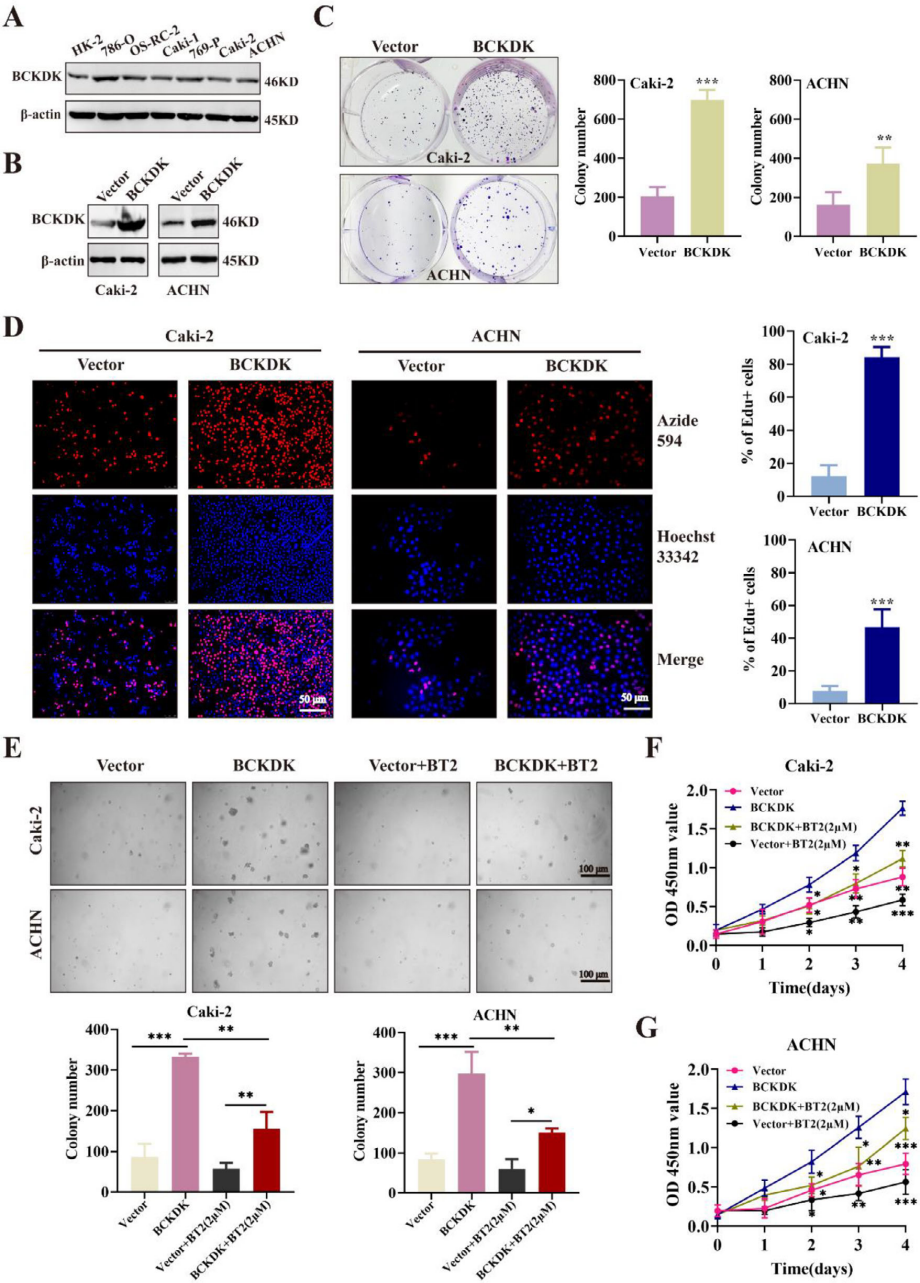
BCKDK promotes RCC cell proliferation in vitro. A) BCKDK expression in HK2 and six RCC cell lines. B) Overexpression of BCKDK in Caki‐2 and ACHN cells. C) Plate colony formation images and colony count statistics of BCKDK‐overexpressing RCC cells. D) Representative fluorescent images showing EdU‐594 positive cells (red), total nuclei (Hoechst 33342, blue), and merged images of EdU‐tagged vector (control) and BCKDK‐overexpressing RCC cells. E) Soft agar assay of BCKDK‐overexpressing RCC cells. Colony numbers were quantified using Graph Prism 9.0.2 software. F) G. CCK‐8 assay detecting cell viability after BCKDK overexpression in RCC cells.

To further elucidate the biological role of BCKDK kinase in RCC progression, in vitro cellular studies were conducted. BCKDK expression levels were analyzed in the renal epithelial cell line HK2 and six RCC cell lines (**Figure** **2**A). Gain‐of‐function experiments were performed by overexpressing BCKDK in Caki‐2 and ACHN cells, which exhibit relatively low BCKDK expression. Overexpression efficiency was confirmed by Western blot and real‐time quantitative polymerase chain reaction (RT‐qPCR) (Figure 2B; Figure S2A, Supporting Information). To assess the impact of BCKDK overexpression on cell proliferation, plate colony formation, and EdU cell proliferation assays were conducted: BCKDK overexpression significantly increased the number of RCC cell colonies (Figure 2C) and promoted cell proliferation (Figure 2D). To evaluate the effect of BCKDK inhibition, the BCKDK inhibitor 3,6‐dichlorobenzo1[b]thiophene‐2‐carboxylic acid (BT2)^[^ ^25^, ^26^ ^]^ was used in soft agar and CCK‐8 assays. BT2 treatment notably reduced colony formation (Figure 2E) and decreased the viability of BCKDK‐overexpressing Caki‐2 and ACHN cells (Figure 2F,G). These results confirmed that upregulation of BCKDK enhances RCC cell proliferation in vitro.

### 2.3 BCKDK Deficiency Inhibits RCC Tumorigenesis

**Figure 3:**
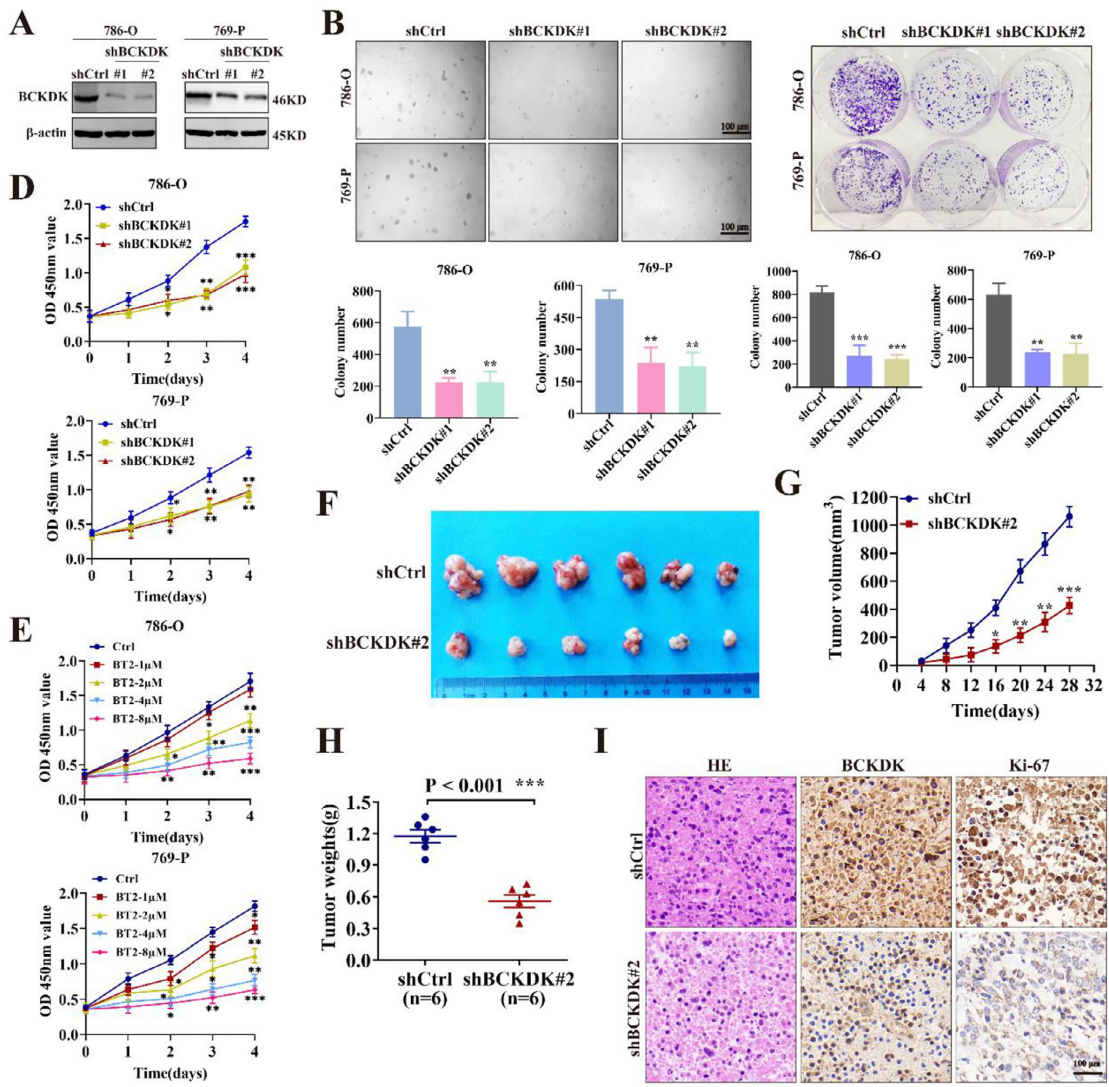
BCKDK deficiency inhibits RCC tumorigenesis. A) Western blot detection of BCKDK knockdown efficiency in RCC cells. B) Soft agar assay of BCKDK knockdown RCC cells. Colony numbers were quantified using Graph Prism 9.0 software. C) Representative images of plate colony formation and colony count statistics of BCKDK knockdown RCC cells. D) CCK‐8 assay measuring cell viability of BCKDK knockdown cells. E) CCK‐8 assay assessing the cell viability of RCC cells treated with BT2. F) Images of subcutaneous xenografts in mice injected with BCKDK knockdown and control 786‐O cells. G) Tumor volume of xenografted tumors from shCtrl and shBCKDK cells. H) Comparison of tumor weight in the shCtrl and shBCKDK groups at the final time point. I) Representative HE and IHC images of BCKDK and Ki‐67 expression in xenografted tumor tissues. Scale bar: 100 µm.

The 786‐O and 769‐P cells, which exhibit relatively high BCKDK expression (Figure 2A), were selected for loss‐of‐function studies. BCKDK expression was knocked down using short hairpin RNA, with knockdown efficiency confirmed by Western blot and RT‐qPCR (**Figure** **3**A; Figure S2B, Supporting Information). Soft agar, plate colony formation, and CCK‐8 assays were subsequently performed to assess the impact of BCKDK deficiency on cell proliferation. Consistent with the gain‐of‐function phenotypes (Figure 2), BCKDK knockdown significantly reduced colony formation and cell viability (Figure 3B–D). Treatment with BT2 led to a dose‐dependent reduction in the viability of RCC cells (Figure 3E).

To explore the role of BCKDK in vivo, subcutaneous injections of BCKDK‐deficient 786‐O and control cells were performed in BALB/c nude mice to assess tumorigenesis. In line with the in vitro findings, BCKDK knockdown markedly inhibited tumor growth in 786‐O cell xenografts (Figure 3F,G). Tumor weight in the BCKDK knockdown group was significantly lower than in the control group (Figure 3H). Moreover, IHC analysis of tumor sections from the mice revealed that BCKDK deficiency significantly reduced Ki‐67 expression (Figure 3I), indicating decreased cell proliferation.

Together, the above results suggested that BCKDK plays a pivotal role in promoting RCC tumorigenesis both in vitro and in vivo.

### 2.4 BCKDK‐Mediated Phosphorylation of AKT Decreases Ubiquitination‐Mediated Degradation, and Promotes Tumorigenesis via Activation of AKT/mTOR Signaling Pathway

Deficiencies in BCAAs catabolism confer functional advantages, which could be exploited for therapeutic interventions in cancers. Given that BCKDK negatively regulates BCKDH activity to control BCAAs catabolism (Figure S3A, Supporting Information), whether BCKDK promote RCC proliferation in a BCAA‐dependent manner? CCK‐8, soft agar, and colony formation assays revealed that BCAAs accumulation did not induce RCC cell proliferation (Figure S3B–D, Supporting Information). Prior studies have shown that BCKDK promotes tumor cell proliferation in CRC by activating the MAPK/ERK signaling pathway,^[^ ^27^ ^]^ leading us to hypothesize a similar role in RCC. However, our data indicate that BCKDK failed to activate the MEK/ERK pathway in RCC cells (Figure S4A,B, Supporting Information).

**Figure 4:**
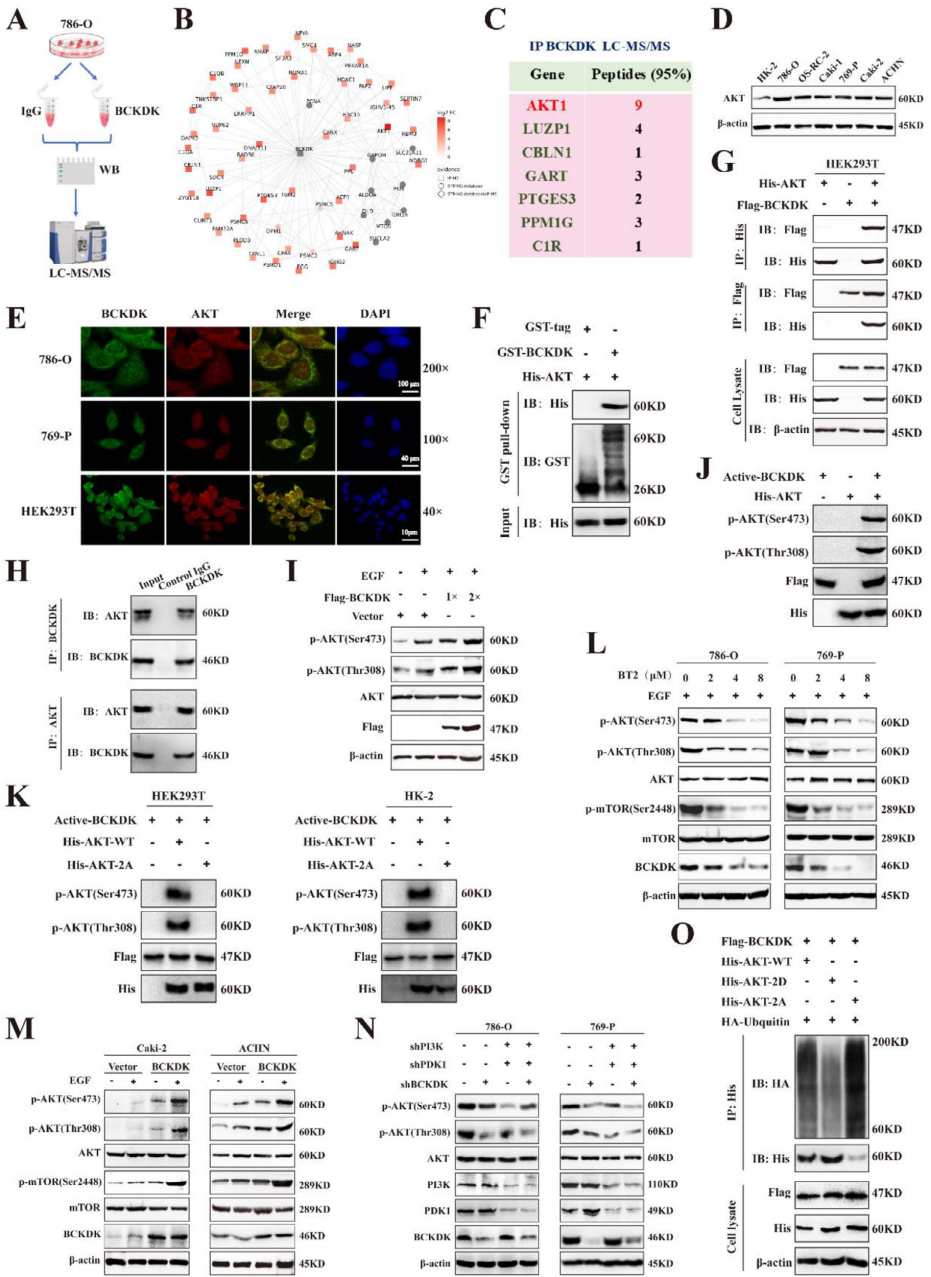
BCKDK‐mediated phosphorylation of AKT decreases ubiquitination‐mediated degradation and promotes tumorigenesis via activation of the AKT/mTOR signaling pathway. A) Flowchart of LC‐MS/MS‐based interactome analysis of BCKDK. B) Protein interaction network diagram, with BCKDK as the central node. C) The protein and peptides in the LC‐MS/MS dataset are shown. D) AKT expression in renal epithelial cell line HK2 and various RCC cell lines. E) Confocal microscopy visualization of BCKDK and AKT colocalization in cells. F) Western blot analysis for the binding of BCKDK and AKT in GST pull‐down assays. G,H) Coimmunoprecipitation of exogenous or endogenous BCKDK with AKT in cells. I) Dose‐dependent promotion of AKT phosphorylation by BCKDK (EGF, 30 ng mL^−1^, 10 min). J) Flag‐BCKDK was transfected into HEK293T cells, and active BCKDK kinase was acquired through IP assay with Flag antibody, followed by His‐AKT as substrate in the in vitro kinase assay. K) In vitro kinase assay detecting p‐AKT phosphorylation signals on downstream His‐AKT‐WT or His‐AKT‐2A substrates, with BCKDK acting as the upstream kinase. L) The effect of BCKDK inhibition on p‐AKT and p‐mTOR expression was measured (EGF, 15 min, 20 ng mL^−1^). M) The p‐AKT and p‐mTOR expression in BCKDK overexpression RCC cells (EGF, 15 min, 20 ng mL^−1^). N) Western blot analysis of PI3K and PDK1 expression effects on BCKDK‐mediated AKT phosphorylation. O) The ubiquitination level of AKT was detected in HEK293T cells transfected with Flag‐BCKDK, His‐AKT‐2A/2D, and HA‐Ubquitin plasmids.

To further investigate the signaling networks of BCKDK, BCKDK was purified from RCC cell lysates via immunoprecipitation and analyzed by LC‐MS/MS (**Figure** **4**A). A protein interaction network was constructed by integrating IP‐MS/MS with the STRING database (Figure 4B). Analysis of peptides and proteins identified from the BCKDK IP group (≥4 fold change compared to control) revealed 1039 potential interactors, with AKT1 exhibiting the highest fold change (Figure 4C; Table S1, Supporting Information). Correlation analysis of co‐expressed genes in RCC from TCGA showed that high BCKDK expression correlates with elevated expression of AKT family members, including AKT1, AKT2, and AKT3 (Figure S5A, Supporting Information). AKT expression was assessed, and immunofluorescence assays demonstrated that BCKDK colocalized with AKT (Figure 4D,E). GST pull‐down and Co‐immunoprecipitation assays indicated that BCKDK interacts with AKT (Figure 4F–H).

As a serine/threonine kinase, BCKDK might directly phosphorylate AKT to regulate its function. To test this, transient transfection and in vitro kinase assays were performed; the results showed that BCKDK mediates phosphorylation of AKT at both Ser473 and Thr308 sites (Figure 4I,J). Additionally, mutant AKT (S473A and T308A, referred to as AKT‐2A) was generated; the phosphorylation level of AKT‐WT was significantly higher than that of AKT‐2A (Figure 4K). As shown in Figure 4L,M, BT2 treatment resulted in a dose‐dependent downregulation of p‐AKT and the downstream target p‐mTOR, while BCKDK overexpression exhibited the opposite effects.

Analysis of subcutaneous tumor tissues from mice demonstrated that BCKDK silencing inhibited AKT/mTOR pathway activation in vivo (Figure S5B, Supporting Information). Elevated BCKAs level failed to promote AKT phosphorylation levels in RCC cells (Figure S5C, Supporting Information), contrasting with observations in metabolic disorders.^[^ ^28^ ^]^ This more definitively demonstrates that BCKDK mediates RCC progression through a mechanism independent of BCAAs/BCKAs accumulation. Besides, in BCKDK‐overexpressing or knockdown cell lines, the mRNA expression level of miR‐125a‐5p was not affected by the expression level of AKT (Figure S5D,E, Supporting Information). Given that previous studies have shown that AKT activation at Thr308 and Ser473 is primarily regulated by upstream kinases PDK1^[^ ^29^ ^]^ and PI3K,^[^ ^30^, ^31^ ^]^ respectively, the involvement of PDK1 and PI3K in BCKDK‐mediated phosphorylation and activation of these AKT sites in RCC was investigated. Whereas, no significant difference in the levels of p‐AKT between the BCKDK knockdown and triple knockdown groups, suggesting that phosphorylation of AKT mediated by BCKDK is independent of PDK1/PI3K expression (Figure 4N). Besides, the ubiquitination level of AKT‐2A (constitutively nonphosphorylated) was more pronounced than that of both the AKT‐WT and AKT‐2D (mimic phosphorylated) (Figure 4O). Treatment with either E3 ligase inhibitor MLN4924 or proteasome inhibitor MG132 substantially reduced AKT‐2A ubiquitination (Figure S6A,B, Supporting Information). Notably, compared to control groups, expression of either Flag‐BCKDK‐WT or His‐AKT‐2D significantly extended AKT protein half‐life (t_1/2_) (Figure S6C,D, Supporting Information).

Collectively, these findings demonstrated that BCKDK‐mediated phosphorylation confers protection against ubiquitin‐proteasome‐mediated degradation of AKT, thereby enhancing its stability, and promotes RCC tumorigenesis via the activation of the AKT/mTOR signaling pathway.

### 2.5 BCKDK/AKT/ABCB1 Axis Mediates Doxorubicin Cytotoxicity in RCC

**Figure 5:**
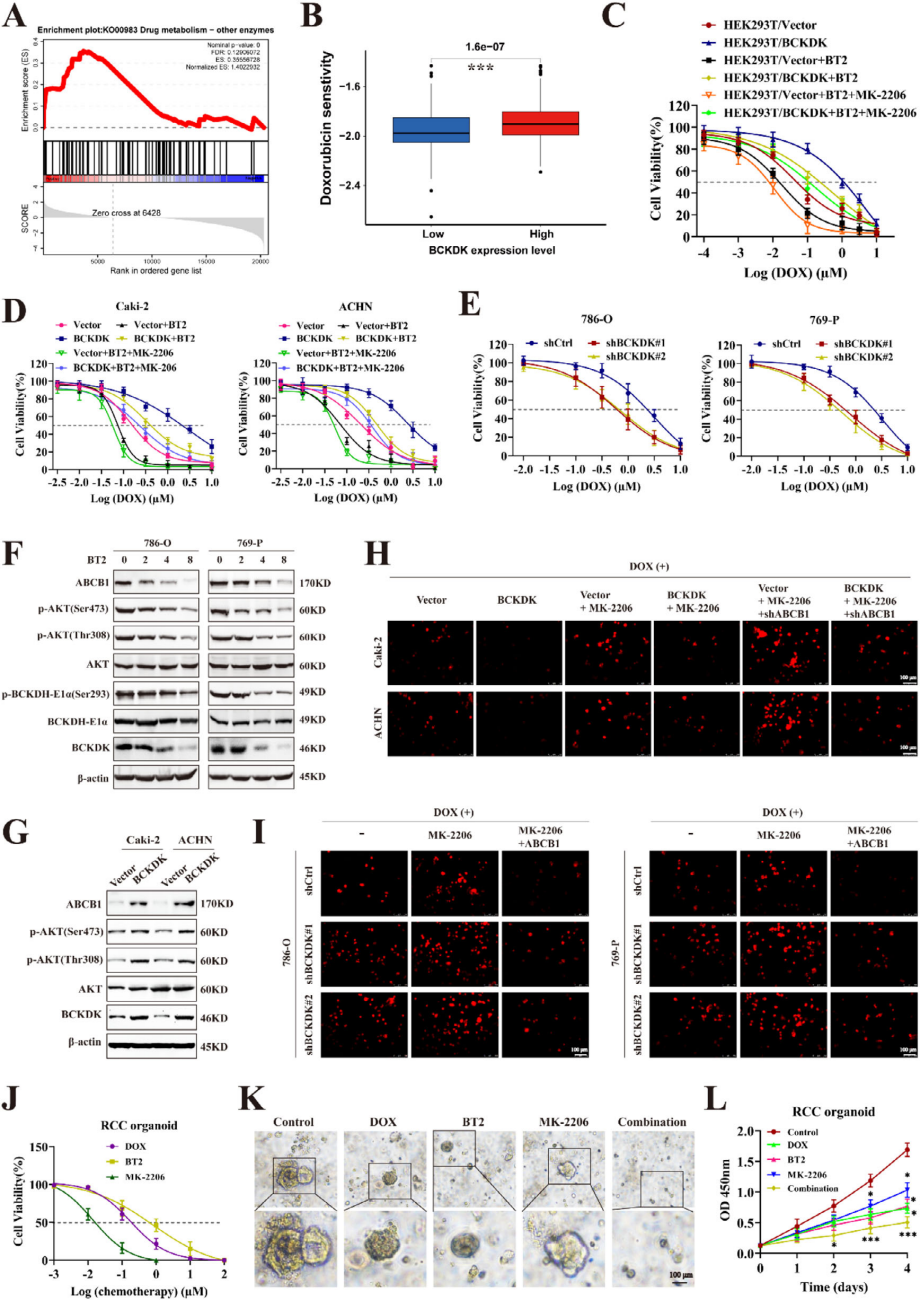
BCKDK/AKT/ABCB1 axis mediates doxorubicin cytotoxicity in RCC cells. A) Gene set enrichment analysis (GSEA) revealed upregulation of drug metabolism signaling pathways in BCKDK knockdown RCC cells. B) Correlation between BCKDK expression and DOX sensitivity in RCC was analyzed using the TCGA database. C) Cell viability of BCKDK overexpressing cells with or without inhibitor treatment. D) BCKDK‐overexpressing RCC cells were treated with or without BT2 and MK‐2206, and DOX cytotoxicity was evaluated. E) DOX cytotoxicity in BCKDK knockdown RCC cells. F) Expression levels of p‐BCKDH, p‐AKT, and ABCB1 were analyzed in RCC cells treated with BT2 at various concentrations. G) Expression levels of p‐AKT and ABCB1 in BCKDK‐overexpressing cells. H) Intracellular DOX accumulation in BCKDK‐overexpressing cells with or without MK‐2206 treatment or ABCB1 knockdown was determined after treatment with 0.5 µM DOX. I) After treatment with 1 µM DOX, DOX residue levels were assessed in BCKDK knockdown cells with or without MK‐2206 treatment and ABCB1 overexpression. J) Dose‐response curves of DOX, BT2, and MK‐2206 of RCC organoids. K) Representative images of DOX, BT2, MK‐2206, and combination (all three drugs) drug sensitivity of RCC organoids. The drug concentrations of DOX, BT2, and MK‐2206 were 1,4, and 50 nM, respectively. L) CCK‐8 assay assessing the viability of RCC organoids treated with DOX, BT2, MK‐2206, and a combination of all three drugs.

To comprehensively evaluate the role of BCKDK in RCC, RNA‐Seq analysis was performed on BCKDK knockdown cells. Gene set enrichment analysis revealed that differentially expressed genes were enriched in various signaling pathways, including the drug metabolism network (**Figure** **5**A). As indicated by the TCGA cytotoxicity profile, DOX was less effective in BCKDK‐high‐expressing KIRC cells (Figure 5B). BCKDK overexpressing cells exhibited significantly greater resistance to DOX, while combined treatment with BT2 and AKT inhibitor MK‐2206^[^ ^32^ ^]^ enhanced DOX‐induced cytotoxicity; Conversely, BCKDK knockdown cells displayed increased sensitivity to DOX (Figure 5C–E).

Various ABC transporters, particularly ATP‐binding cassette B1/P‐glycoprotein (ABCB1/P‐gp, MDR1) and breast cancer resistance protein (ABCG2), have been implicated in tumor chemotherapy resistance. These transporters are upregulated in numerous tumors and actively extrude a broad range of chemotherapeutic agents, making them promising therapeutic targets.^[^ ^33^, ^34^ ^]^ ABCB1 is one of the most extensively studied ABC transporters, with a wide substrate profile that includes taxanes, vinca alkaloids, DOX, and various tyrosine kinase inhibitors (TKIs).^[^ ^35^ ^]^ In acute myeloid leukemia, GSPE‐regulated multidrug resistance may be associated with suppression of the PI3K/AKT pathway, leading to decreased ABCB1 expression.^[^ ^36^ ^]^ Based on these observations, it was hypothesized that the activation of the BCKDK/AKT/ABCB1 signaling cascade contributes to DOX resistance in RCC. As shown in Figure 5F, BT2 significantly decreased the expression of p‐AKT and ABCB1. Treatment with MK‐2206 resulted in a significant reduction in ABCB1 (Figure S7A, Supporting Information). BCKDK overexpression led to a notable increase in the expression of p‐AKT and ABCB1 (Figure 5G). MK‐2206 treatment downregulated ABCB1 expression (Figure S7B, Supporting Information). Docking simulation results showed that BT2 docked into the inhibitory binding site of ABCB1 with an affinity score of −8.228 kcal mol^−1^. Details of ligand‐receptor interaction are displayed in Figure S7C,D (Supporting Information). We can observe the *π–π* interactions at residues Tyr310, Phe335, and Phe314. Moreover, Phe314 also forms an H‐bond with BT2. The molecular docking indicated that BT2 exhibited a high docking score toward ABCB1 at the binding site, suggesting strong affinities with ABCB1, which explains the phenomenon that BT2 sensitized RCC cells to doxorubicin through competitive inhibition of the ABCB1 protein.

Subsequently, the rescue experiments revealed that ABCB1 knockdown, in combination with MK‐2206 treatment, significantly increased DOX accumulation in BCKDK‐overexpressing cells (Figure 5H). In contrast, MK‐2206 treatment upregulated DOX retention in BCKDK knockdown cells, while combining MK‐2206 treatment with ABCB1 overexpression notably reduced DOX accumulation in these cells (Figure 5I). Patient‐derived organoids (PDOs) can accurately preserve and recapitulate the genetic landscape and histopathological characteristics of their original tumors, indicating their potential to predict patient treatment responses in personalized cancer therapy.^[^ ^37^, ^38^ ^]^ Drug sensitivity testing of RCC PDOs revealed that the combination of DOX with BT2 and MK‐2206 could maximally inhibit the growth rate of PDOs (Figure 5J–L). These results underscored that BCKDK/AKT/ABCB1 mediates DOX cytotoxicity in RCC.

### 2.6 Targeting BCKDK Promotes Doxorubicin‐Mediated Apoptosis and Inhibits RCC Tumorigenesis

**Figure 6:**
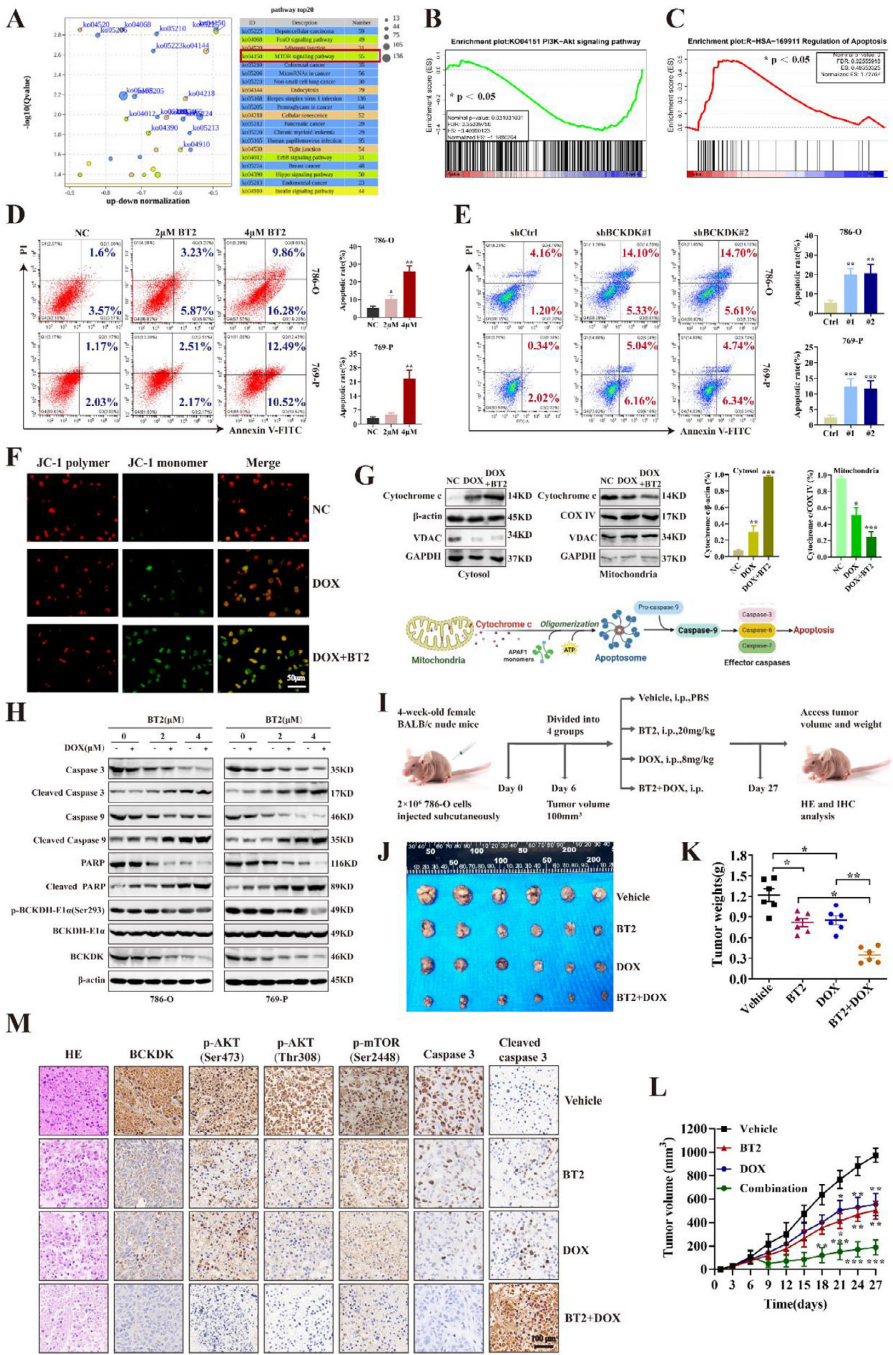
Targeting BCKDK promotes doxorubicin‐mediated apoptosis and inhibits RCC tumorigenesis. A) Functional enrichment‐based clustering analysis of the KEGG pathways involved in BCKDK‐related signaling. The top 20 pathways are shown. B) GSEA shows that differentially expressed genes are enriched in the PI3K/AKT signaling network. C) GSEA indicates an upregulation of the apoptosis signaling pathway in BCKDK knockdown RCC cells. D,E) Apoptosis of RCC cells treated with BT2 at different concentrations and BCKDK knockdown cells was detected by flow cytometry. The corresponding bar graphs represent statistical results from three independent replicate experiments, and the quantified data include both Annexin V and PI double‐positive cells (late apoptotic cells) and Annexin V single‐positive cells (early apoptotic cells). F) The mitochondrial membrane potential assay kit with JC‐1 was used to assess changes in mitochondrial membrane potential in 786‐O cells treated with DOX alone or in combination with BT2. G) The expression levels of cytochrome c in both cytosolic and mitochondrial fractions of RCC cells were detected after drug treatment, with β‐actin/GAPDH and COX IV/VDAC serving as internal controls for normalization, respectively. The flowchart below illustrates the mechanistic pathway of mitochondria‐mediated apoptosis, showing the release of pro‐apoptotic factor cytochrome c and subsequent activation of caspase‐9, which further triggers downstream effector caspases to execute apoptotic effects. H) RCC cells were treated with 0, 2, and 4 µM BT2 for 24 h, followed by 2 µM DOX or DMSO treatment for 12 h. The effect of DOX‐mediated cell death and apoptosis was analyzed. I) 786‐O cells were used to develop subcutaneous xenografts in BALB/c nude mice. J) The picture of dissected subcutaneous xenografts. K) Tumor weight statistics at the point of final time. L) Tumor volume of each group in mice. M) HE staining and representative IHC images in xenografted tumors.

RNA‐Seq analysis revealed that BCKDK deficiency was associated with dysregulation of several transcription factors, with the mTOR signaling pathway ranking among the top 20 affected pathways (**Figure** **6**A), consistent with our findings in Figure 4 and Figure S5 (Supporting Information). Differentially expressed genes were enriched in multiple signaling networks, including the PI3K/AKT and apoptosis pathways (Figure 6B,C). The Annexin V‐FITC/PI double staining demonstrated that BCKDK inhibition or knockdown induced apoptosis of RCC cells (Figure 6D,E). Treatment with the MK‐2206 alone moderately induced apoptosis in RCC cells, whereas combined treatment with the MK‐2206 and BT2 significantly enhanced RCC cell apoptosis (Figure S8A,B, Supporting Information). MK‐2206 exhibited no significant effect on mitochondrial membrane potential, whereas DOX or BT2 alone could perturb it. Notably, the DOX/BT2 combination and DOX/BT2/MK‐2206 triple combination induced the most dramatic reduction in mitochondrial membrane potential in RCC cells (Figure 6F; Figure S8C, Supporting Information), suggesting BCKDK plays a pivotal role in the early phase of RCC apoptosis.

In the intrinsic apoptosis pathway, pro‐apoptotic proteins of the BCL‐2 family integrate into the mitochondrial outer membrane and oligomerize to form pores, enabling the release of pro‐apoptotic factors from the mitochondrial intermembrane space into the cytosol.^[^ ^39^ ^]^ Accordingly, cytosolic and mitochondrial fractions were isolated from drug‐treated RCC cells, and the highest cytochrome c release was detected in the DOX/BT2 combination group (Figure 6G, upper panel). These results suggest BCKDK's involvement in the mitochondria‐mediated pathway where cytochrome c binds to apoptotic protease‐activating factor‐1 and then recruits caspase‐9 monomers, inducing their dimerization and proteolytic activation. Activated caspase‐9 subsequently cleaves downstream executioner caspases‐3, −6, or −7 to execute apoptosis (Figure 6G, lower panel). As shown in Figure 6H, at lower concentrations, BT2 modestly enhanced DOX‐induced expression of cleaved caspase 3, cleaved caspase 9, and cleaved PARP, with maximal effects observed at 4 µM, indicating that targeting BCKDK promotes mitochondria‐mediated cytochrome c release and caspase‐9 activation, which subsequently triggers the downstream executioner caspase‐3 to enhance RCC cell apoptosis.

Subcutaneous tumor transplantation experiments in mice further confirmed that combining BT2 with DOX maximized tumor inhibition in vivo (Figure 6G–L). IHC analysis of transplanted tumors revealed that BCKDK, p‐AKT, p‐mTOR, and caspase‐3 expression were significantly downregulated in BCKDK‐deficient tumors (Figure 6M), indicating that targeting BCKDK enhances doxorubicin‐mediated apoptosis and suppresses tumorigenesis in RCC.

Collectively, these findings highlight the critical role of BCKDK activation in RCC tumor progression. What mechanisms underlie BCKDK activation? Our previous study found that Src could activate BCKDK in CRC, promoting tumor malignancy.^[^ ^22^ ^]^ However, knockdown and inhibition of Src did not affect BCKDK activity of RCC cells (Figure S9A,D, Supporting Information). Suggesting that the upstream factors mediate BCKDK activation in RCC requires further investigation.

**Figure 7:**
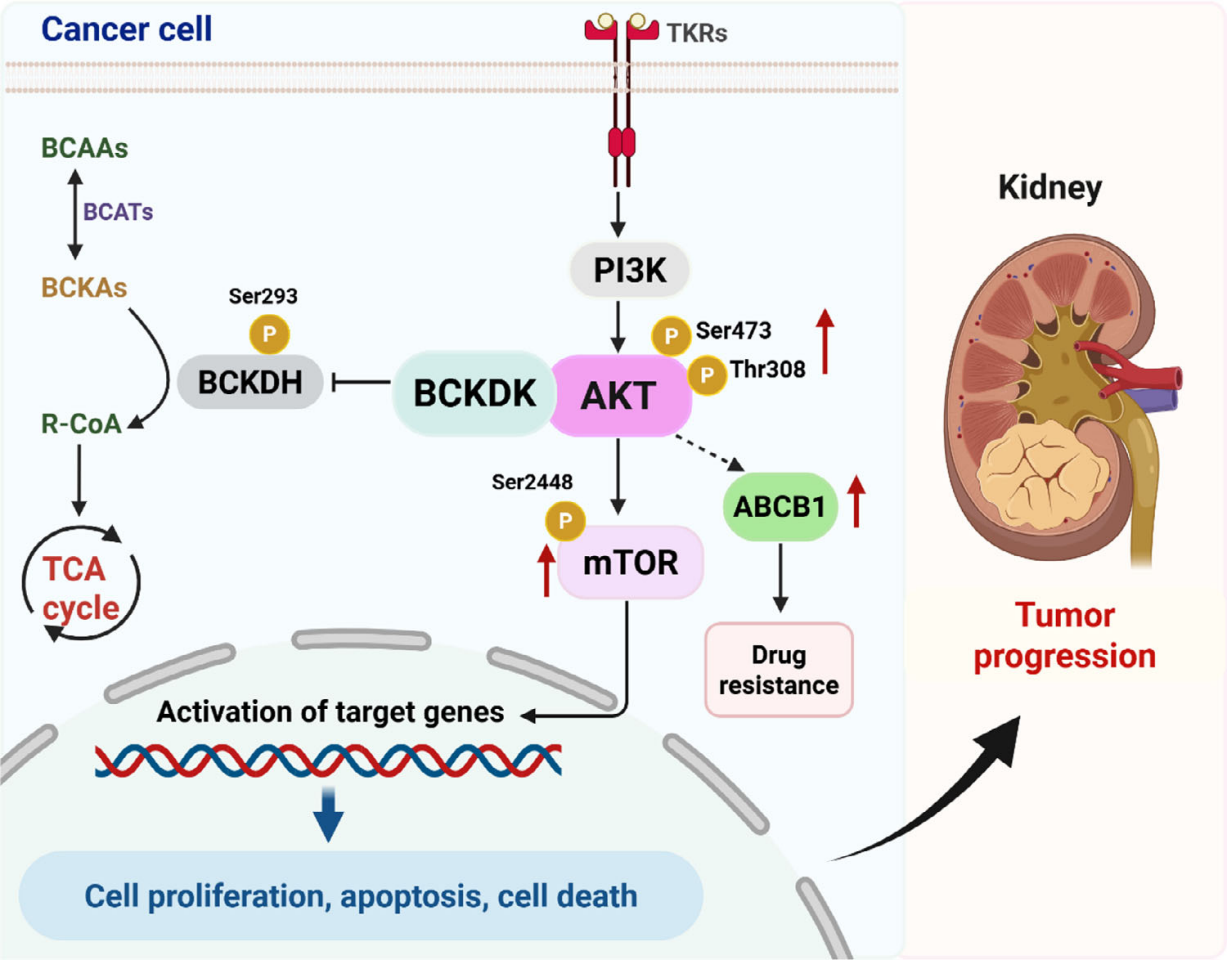
Schematic diagram of BCKDK promoting RCC progression via AKT‐related signaling pathways.

In conclusion, our studies revealed that BCKDK directly binds to and modulates AKT phosphorylation at both Ser473 and Thr308 sites, activating the downstream AKT/mTOR and AKT/ABCB1 signaling pathways, thereby promoting tumorigenesis, apoptosis, and drug resistance of RCC cells (**Figure** **7**). Scientific illustrations were created with BioRender.com.

## 3 Discussion

The role of BCAAs metabolism in human tumors presents contradictory findings. In human HCC, inhibition of BCAAs catabolic enzymes resulted in BCAAs accumulation, and progressive deficiency in BCAAs catabolism contributed to cancer development.^[^ ^19^ ^]^ However, elevated BCAA levels have also been closely associated with an increased risk of PDAC.^[^ ^13^ ^]^ Another study found that elevated plasma BCAAs levels were observed in early‐stage PDAC but not in NSCLC, despite the cancers being triggered by the same genetic events.^[^ ^40^ ^]^ In contrast, BCAAs supplementation did not promote the proliferation of RCC cells. Instead, the key BCAAs catabolic enzyme BCKDK was upregulated in RCC tissues and predicted a negative prognosis for patients with RCC in this study. Previous research has shown that BCKDK plays a critical role in RCC metastasis,^[^ ^41^ ^]^ but the specific mechanisms behind this remain underexplored. Therefore, further studies are needed to investigate the relationship between BCAAs metabolism and BCKDK kinase activity in the development of tumors.

Over the past decade, much of the research on cancer‐related BCAAs metabolism has focused on branched‐chain amino acid transaminase 1 and 2 (BCAT1/2), which play essential roles in the pathogenesis of various cancer types, including AML,^[^ ^11^ ^]^ GBM,^[^ ^18^ ^]^ gastric cancer,^[^ ^42^ ^]^ and PDAC.^[^ ^13^, ^14^ ^]^ These enzymes are considered potential therapeutic targets. For example, BCAT2 has been identified as a key suppressor of ferroptosis, which could inform the responsiveness to ferroptosis‐inducing therapies in HCC, CRC, and fibrosarcoma cells.^[^ ^43^ ^]^ BCAT2 has also been implicated in forming a non‐inflammatory tumor microenvironment (TME) in bladder cancer. Co‐treatment with anti‐PD‐1 antibody and BCAT2 deficiency exhibited a combined effect in vivo, suggesting BCAT2 as a potential target for combination therapies involving immune checkpoint blockade (ICB).^[^ ^44^ ^]^ Interestingly, BCAT2 binds to the BCKDH complex, and both BCAT2 binding and BCKDK phosphorylation compete for the same site on the complex. BCAT2 binding upregulates BCKDH activity, while BCKDK phosphorylation disrupts the interaction between BCKDH and BCAT2.^[^ ^45^ ^]^ Moreover, recent studies have reported that BCAT1 and its newly identified E3 ubiquitin ligase are phosphorylated by BCKDK. This cross‐regulation between BCKDK‐dependent phosphorylation and STUB1‐mediated ubiquitination accelerates GBM progression.^[^ ^46^ ^]^ These findings underscore the complexity of BCAA metabolism in cancer and highlight the need for further studies to understand the reciprocal regulatory relationships between BCAA metabolic enzymes. Such research could lead to the development of novel therapeutic targets for cancer treatment.

Extensive evidence underscores the pivotal role of BCKDK beyond regulating BCAA pools in tumor progression. APN‐mediated phosphorylation of BCKDK at Ser31 activates the downstream MEK/ERK signaling pathway, driving tumorigenesis and metastasis in HCC.^[^ ^21^ ^]^ Inhibition of BCKDK impairs mitochondrial function and protein synthesis, rendering triple‐negative breast cancer (TNBC) more susceptible to cell death.^[^ ^47^ ^]^ BCKDK knockout in NSCLC cell lines, as demonstrated by Wang et al., suggests that BCKDK facilitates tumor progression by modulating the tricarboxylic acid (TCA) cycle and glycolysis.^[^ ^48^ ^]^ Recent studies have shown that BCKDK phosphorylates the pyruvate dehydrogenase complex (PDHC), resulting in pyruvate deficiency and disruption of the TCA cycle.^[^ ^49^ ^]^ BCKDK activates the MAPK pathway through direct phosphorylation of MEK at Ser221, contributing to tumorigenesis in CRC.^[^ ^27^ ^]^ Elevated BCKDK levels correlate with CRC metastasis and poor prognosis in patients.^[^ ^22^ ^]^ In contrast, our data reveal that BCKDK does not activate the MEK/ERK signaling pathway in RCC, contradicting prior findings in ccRCC,^[^ ^24^ ^]^ warranting further investigation.

Preliminary studies revealed that the BCKDK/exosomal‐miR‐125a‐5p/VE‐cadherin signaling axis mediates intercellular communication between HUVECs and RCC cells to regulate the vascular microenvironment.^[^ ^24^ ^]^ However, our present study specifically focuses on BCKDK's protein kinase function, providing an in‐depth investigation into how its tumor‐associated overexpression mediates tumorigenesis and drug resistance through phosphorylation‐dependent regulation of downstream substrates. In this study, a novel mechanism is proposed, where BCKDK promotes tumorigenesis in RCC by directly mediating downstream AKT phosphorylation at Ser473 and Thr308, thereby activating the AKT/mTOR pathway. Notably, our findings suggest that in RCC progression, the BCKDK/AKT/mTOR signaling axis‐mediated pro‐tumor mechanisms represent a molecular pathway distinct from the previously reported BCKDK‐regulated exosomal miR‐125a‐5p/VE‐cadherin axis that activates angiogenesis to facilitate tumor metastasis. The underlying reasons for this mechanistic divergence await further investigation.

RNA sequencing data revealed that BCKDK is closely associated with the regulation of drug metabolism networks and apoptotic signaling pathways. Mechanistic investigations demonstrated that the BCKDK/AKT/ABCB1 axis mediates DOX sensitivity in RCC. Targeted inhibition of the BCKDK/AKT significantly enhanced DOX‐induced apoptosis in RCC cells and suppressed tumor growth in vivo. Our study not only identified novel downstream phosphorylation substrates of BCKDK but also elucidated the critical role of the BCKDK/AKT axis in RCC progression, providing a potential therapeutic target for clinical intervention in cancer. Consistent with previous reports, BCKDK's role as a protein kinase in tumor progression warrants focused attention and further investigation. As explicitly highlighted, BCKDK represents a promising novel kinase target with potential applications in metabolic and oncological diseases. Whereas the precise mechanism underlying BCKDK‐mediated phosphorylation of AKT at Ser473 and Thr308 remains unclear, necessitating further research. Transcriptomic analysis indicates that BCKDK downregulation alters multiple biological processes, including mTOR, ErbB, Hippo, and apoptosis signaling pathways, highlighting its critical role in RCC tumor progression. Furthermore, BCKDK‐mediated AKT phosphorylation in RCC is independent of PI3K and PDK1 expression levels. Whether this holds true in other tumor types remains uncertain, and future studies utilizing PI3K and PDK1 knockout cancer cell lines or animal models are needed to explore this further.

Inactivation of the *von* H*ippel‐Lindau* (*VHL*) gene is commonly observed in clear‐cell renal cell carcinoma (ccRCC), with ≈70% of ccRCC cases harboring mutations in the *VHL* gene, which encodes the VHL protein (pVHL).^[^ ^50^ ^]^ These mutations lead to the inactivation of pVHL, causing the activation and accumulation of hypoxia‐inducible factors (HIFs), which are central to RCC pathogenesis due to their role in promoting cancer cell proliferation, glucose metabolism, angiogenesis, metastatic spread, and disease progression.^[^ ^51^, ^52^ ^]^ Clinical trials targeting hypoxia‐related signaling molecules are currently underway as potential therapeutic strategies for tumors.^[^ ^53^, ^54^ ^]^ Drugs targeting the pVHL/HIF/vascular endothelial growth factor (VEGF) signaling axis have demonstrated efficacy in treating RCC and are now considered the standard therapy for metastatic cases.^[^ ^55^ ^]^ Furthermore, suppression of BCAT1 has been shown to impair GBM cell growth under hypoxic conditions by disrupting HIF‐mediated BCAAs metabolism.^[^ ^56^ ^]^ The mechanisms by which VHL promotes RCC progression are complex, particularly in relation to metabolism, with amino acid metabolism emerging as a critical area of interest. Given their pivotal roles in regulating BCAAs metabolism, the interactions between BCAT1/2, BCKDK, VHL, and HIF warrant further investigation.

Drug resistance remains a significant challenge in RCC chemotherapy,^[^ ^57^, ^58^, ^59^ ^]^ necessitating the exploration of new strategies to enhance drug sensitivity. ABCB1, one of the most well‐characterized human efflux transporters, is predominantly expressed in the intestine, kidney, liver, and blood‐brain barrier.^[^ ^60^ ^]^ Activation of the AKT signaling pathway contributes to ABCB1 expression and function in cisplatin‐resistant NSCLC cells.^[^ ^61^ ^]^ In TNBC, DOX has been shown to regulate BCAA metabolism, implicating BCKDK as a DOX‐sensitive target,^[^ ^47^ ^]^ although the exact mechanism remains unclear.

Our findings demonstrate that activation of the BCKDK/AKT pathway upregulates ABCB1 expression and reduces DOX sensitivity in RCC cells. Conversely, BCKDK deficiency enhances cell death and promotes DOX‐induced apoptosis, which aligns with observations in TNBC.^[^ ^47^ ^]^ Another study in TNBC similarly found increased apoptosis upon BCKDK knockdown, though not in a chemoresistance context.^[^ ^62^ ^]^ Notably, our study reveals fundamental differences in BCKDK‐mediated apoptotic mechanisms compared to those reported in TNBC. While BCKDK‐dependent apoptosis in TNBC relies on the terminal executioner caspase‐7,^[^ ^47^ ^]^ we demonstrate that BCKDK targeting in RCC activates the mitochondrial apoptotic pathway, triggering cytochrome c release and subsequent activation of caspase‐9, which further activates the downstream executioner caspase‐3, ultimately inducing apoptosis. Suggesting that BCKDK participates in cell death regulation through distinct apoptotic pathways across different tumor types. By elucidating the molecular mechanisms underlying DOX resistance in RCC, our study provides a theoretical foundation for developing optimized DOX‐based combination therapies.

Overall, this study identifies a novel oncogenic role for BCKDK in RCC, wherein it promotes tumor progression and drug resistance through AKT phosphorylation and activation of AKT‐related signaling pathways. These findings offer a potential prognostic marker and therapeutic target for RCC.

## 4 Experimental Section

### Cell Culture, Plasmids, and shRNAs

Human OS‐RC‐2, 786‐O, ACHN, 769‐P, Caki‐2, Caki‐1, and HEK293T cells were obtained from the National Collection of Authenticated Cell Cultures (Shanghai Cell Bank, China). Human HK‐2 cells were purchased from Procell Life Science & Technology Co., Ltd. (Wuhan, China). HEK293T, HK‐2, OS‐RC‐2, and ACHN cells were cultured in high‐glucose DMEM (Gibco, USA) supplemented with 10% FBS, 100 units mL^−1^ penicillin (Invitrogen, Carlsbad, CA, USA), and 100 mg mL^−1^ streptomycin (Invitrogen, Carlsbad, CA, USA). 769‐P and 786‐O cells were maintained in 1640 medium (Gibco, USA) containing 10% FBS. Caki‐2 and Caki‐1 cells were cultured in MyCoy’5A medium (Gibco, USA) with 10% FBS. Cell transfection was carried out using Lipofectamine 3000 (Invitrogen, USA). The pDONR223‐BCKDK (catalog: 23794), pDONR223‐AKT1‐WT (catalog: 81764), and pENTR‐ABCB1 (catalog: 221424) plasmids were sourced from Addgene (USA). The shBCKDK sequences are: CCGGTCAGGACCCATGCACGGCTTTCTCGAGAAAGCCGTGCATGGGTCCTGATTTTTG; CCGGACGCTGACTTCGAGGCTTGGACTCGAGTCCAAGCCTCGAAGTCAGCGTTTTTTG. The shCtrl sequence is: CCGGCCTAAGGTTAAGTCGCCCTCGCTCGAGCGAGGGCGACTTAACCTTAGGTTTTTG. The shPI3K sequence is: ATTCACAGATAGCATCTGAT; The shPDK1 sequence is: CGGATCAGAAACCGACACAAT. The shSrc sequences are: CCGGGACAGACCTGTCCTTCAAGAACTCGAGTTCTTGAAGGACAGGTCTGTCTTTTTG; CCGGGTCATGAAGAAGCTGAGGCATCTCGAGATGCCTCAGCTTCTTCATGACTTTTTG.

### Antibodies and Reagents

BCKDK (Santa Cruz, USA, sc‐374424). Phospho‐BCKDK (Y246)^[^ ^22^ ^]^ was presented by Prof. Qiuhong Duan from the Department of Biochemistry and Molecular Biology of the School of Basic Medical Sciences of Huazhong University of Science and Technology (Wuhan, China). Phospho‐mTOR‐(Ser2448) (for IHC, ZRB1553, Sigma). Phospho‐AKT (Thr308) (for IHC, 44–602G, Thermo). Flag (F1804, F7425, Sigma). Cleaved caspase 3 (25128‐1‐AP, Proteintech). Caspase 9 Polyclonal antibody (10380‐1‐AP, Proteintech). PDK1 (18262‐1‐AP, Proteintech). Src (60315‐1‐Ig, Proteintech). VDAC (Santa Cruz, sc‐390996). EGF (MCE). Puromycin and G418 were purchased from Sigma–Aldrich. Valine, leucine, and isoleucine (Sigma, 72‐18‐4 and 1509‐34‐8). BT2 (MCE, 34576‐94‐8). Doxorubicin (MCE, 23214‐92‐8). MK‐2206 (Selleck, 1032350‐13‐2). Dasatinib (Sigma, 302962‐49‐8). The following antibodies were obtained from Cell Signaling Technology: β‐Actin (4970), AKT (4685), p‐AKT (Thr308) (13038), p‐AKT (Ser473) (4060), mTOR (2983), BCKDH‐E1α (90198), phospho‐BCKDH‐E1α (Ser293) (40368), Caspase 3 (9662), Cleaved PARP (9541), p‐mTOR (Ser2448) (5536), Cleaved Caspase‐9 (9505), PARP (9542), ABCB1 (13342), Ki‐67 (8D5) (9449S), Mouse IgG (7076), Rabbit IgG (7074), His (12698), MEK1/2 (9122), p‐MEK1/2 (Ser221) (9154S), ERK1/2 (4695S), p‐ERK1/2 (Thr202/Tyr204) (4370S), PI3 Kinase p110α (C73F8) (4249), COX IV (4850), Cytochrome c (4272), HA (3724), GST (2622), GAPDH (2118). BCKAs:  α‐ketoisocaproate (α‐KIC) (MCE, 4502‐00‐5), α‐Ketoisovaleric acid (KIV) (Sigma, 198994), α‐Keto‐β‐methylvaleric acid (KMV) (Sigma, K7125).

### Western Blot

Cell samples were lysed using RIPA lysis buffer (Millipore, 20–188), and protein concentration was determined via BCA assay (Thermo, A55860). The protein samples were boiled, resolved on 7%–20% SDS‐PAGE gels, and transferred to PVDF membranes (Millipore, USA). Membranes were blocked with 5% nonfat dry milk and incubated overnight at 4 °C with the respective primary antibody. Densitometric analysis was performed via chemiluminescence (Bio‐Rad, USA).

### Co‐IP

Human HEK293T, 786‐O, or HK2 cells were lysed with Pierce IP buffer (Thermo, 87787). After centrifugation, the supernatant was incubated overnight at 4 °C with primary antibody and protein A/G magnetic agarose beads (Santa Cruz, USA). The antibody‐bead‐protein complexes were washed 3–6 times with lysis buffer, boiled in SDS‐PAGE protein loading buffer (Sigma, S3401), and analyzed by Western blot.

### GST Pull‐Down

GST Pull‐down assays were performed as described previously.^[^ ^63^ ^]^ Briefly, the GST‐BCKDK (Sino Biological, B15‐31G) fusion protein, which was pre‐incubated with glutathione Sepharose beads at 4 °C for 2 h, was co‐incubated with RCC cell lysates at 4 °C for 24 h. Subsequently, the protein complexes were washed three times with ice‐cold lysis buffer and eluted with reduced glutathione. Finally, the interacting proteins were detected by Western blot.

### In Vitro Kinase Assay

The assay was conducted as previously described.^[^ ^46^ ^]^ Briefly, HEK293T cells were transfected with Flag‐BCKDK‐pCMV for 48 h, treated with EGF, and collected for IP of active BCKDK kinase using an anti‐Flag antibody. Inactive His‐AKT‐WT and His‐AKT‐2A proteins were obtained via Glutathione Sepharose. Protein concentration was determined using the Bradford method, and grayscale scanning was performed. Active BCKDK kinase and 4 µg of inactive AKT WT/2A substrates were incubated in 1× kinase buffer (CST, 9802) with 200 µM ATP (CST, 9804) for 3 h at 4 °C. Phosphorylation was assessed by Western blot using p‐AKT (Ser473) and p‐AKT (Thr308) antibodies.

### Ubiquitination Degradation

The HEK293T cells were co‐transfected with Flag‐BCKDK, His‐AKT‐2A/2D, and HA‐Ubiquitin (Addgene, 18712) plasmids. After 48 h, cell samples were collected from each group. Then, immunoprecipitation was performed using the His‐tag antibody. Finally, the expression levels of Flag, His, and HA in both total cell lysates and IP samples were detected by Western blot. As for pharmacological inhibitors of the ubiquitination machinery, the HEK293T cells were transfected with the indicated plasmids, then followed by treatment with MLN4924 (MCE, 905579‐51‐3, 1 µM for 12 h) or MG132(MCE, 133407‐82‐6, 10 µM for 8 h). After 48 h, cell samples were collected from each group.

### Cycloheximide Chase Assays

When RCC cells reached ≈60–70% confluency, the medium was replaced with serum‐free medium for 8 h of culture, followed by the addition of 50 µg mL^−1^ CHX (MCE, 66‐81‐9) to the medium to block new protein synthesis. Then, cell lysates were collected at various time points to detect AKT protein expression levels.

### RT‐qPCR

Total RNA from RCC cell lines was extracted using TRIzol reagent (Sigma, USA) according to the manufacturer's protocol. RNA (1 µg) was reverse‐transcribed using the HiScript II Strand cDNA Synthesis Kit (Vazyme Biotech). Real‐time PCR was performed using SYBR Premix Ex Taq (TaKaRa) on a Real‐Time Thermocycler (CFX96, Bio‐Rad, USA), with β‐actin expression as the control. Primer sequences for BCKDK were: forward: TCCGCTGCCTTCCTTTCATCATTG; reverse: TCGTCCGCCTGGTCCTTGATC.

### Computational Docking Analysis

Computational docking analysis was performed as described previously.^[^ ^64^ ^]^ The BT2 3‐D structure was obtained from PubChem to dock a simulation with a human ABCB1 model. The human ABCB1 protein model (PDB ID:7A6E) was obtained from the RCSB Protein Data Bank.^[^ ^65^ ^]^ Calculations of the docking were achieved in Schrodinger Maestro 14 (*Version* 14.2.118). Ligand preparation was essentially performed with the default protocol. Human ABCB1 protein preparation was conducted to optimize the structure, remove water, and minimize the energy. Subsequently, docking grid center coordinates were determined from the bound ligands provided in the PDB structure, and a grid of 30 Å at the binding pocket of the ABCB1 protein was generated. Glide XP docking was performed, followed by induced‐fit docking (IFD), and it was carried out using the default protocol, and the docking score (kcal/mol) was calculated.

### Click‐iT EdU

Cell proliferation was measured using the BeyoClick EdU Cell Proliferation Kit (C0078S) following the manufacturer's instructions. Images were acquired using the Leica DMi8 imaging system.

### CCK‐8

Cell viability was determined using the Cell Counting Kit‐8 (Invitrogen, #96992) according to the manufacturer's instructions.

### Colony‐Forming

Cells were seeded at 1 × 10^3^ cells per well in 6‐well plates and cultured for ≈2 weeks, with medium changes twice weekly. Colonies were analyzed as previously described.^[^ ^46^ ^]^

### Softagar

The softagar assay was conducted as previously described,^[^ ^27^ ^]^ and images were captured using an Olympus microscope.

### ELISA Assays

Phospho‐Akt (Ser473) ELISA Kit (CST, 80895), Phospho‐Akt (Thr308) Sandwich ELISA Kit (CST, 7252), and Phospho‐mTOR (Ser2448) Sandwich ELISA Kit (CST, 7976) were used for ELISA assays. 100 µL of RIPA‐lysed and BCA‐quantified cell lysates and serially diluted standards (0–1200 pg mL^−1^) were added respectively to the capture antibody‐precoated plate (in triplicate for each sample), followed by incubation at 37 °C for 2 h. After washing with 1 × PBST three times, 100 µL of biotin‐conjugated detection antibody was added and incubated for another 2 h. For biotinylated detection antibodies, further incubate with streptavidin‐HRP for 30 min. Following four times of 1 × PBST washes, 100 µL TMB substrate was added for color development at room temperature in the dark for 20 min, then the reaction was stopped with 50 µL stop solution. The absorbance at 450 nm was measured using a microplate reader and the target protein concentration was calculated based on the standard curve.

### Immunofluorescence (IF)

Immunofluorescence was performed as previously described.^[^ ^22^ ^]^ Briefly, HEK293T, 786‐O, and 769‐P cells were seeded onto coverslips and fixed in 4% paraformaldehyde for 30 min. After washing with PBS, cells were permeabilized with 0.2% Triton X‐100 for 20 min and blocked with 10% goat serum for 1 h. Cells were then incubated overnight at 4 °C with primary antibodies targeting BCKDK and AKT. The following day, cells were incubated in the dark at room temperature for 1 h with Alexa Fluor 488 (Abcam, ab150077) or Alexa Fluor 546 (Invitrogen) secondary antibodies. Chromatin was stained with DAPI (Sigma, 28718‐90‐3). Images were acquired using a ZEISS confocal microscope.

### RCC Organoid Culture and Drug Sensitivity

Freshly collected tissues were immediately placed in tissue storage solution. After tissue mincing, digesting, and filtering, the cell clusters were mixed with matrigel (Corning) and then quickly seeded into a 24‐well plate and incubated at 37 °C for 1 h. Subsequently, 600 µL of complete RCC organoid medium (Mingao Biotechnology, Shenzhen, OptoidCM, M2007) was added into the wells and continued culturing at 37 °C for ≈2 weeks; the medium was refreshed every 4 days. Finally, passaging and drug sensitivity assays were performed.

### Annexin V‐FITC/PI Apoptosis

Flow cytometry (FCM) was employed to assess apoptosis. Tumor cells were seeded in 6 cm dishes at a density of 1 × 10^5^ cells per well and cultured for 24 h. Cells were then pretreated with or without BT2. Apoptosis was detected using the Annexin V‐FITC/PI Apoptosis Detection Kit (Vazyme Biotech, A211‐02). Cells were trypsinized, washed twice with 1× binding buffer or PBS, and resuspended in 1×binding buffer. The suspension was mixed and stained with annexin V‐FITC (5 µL) and PI (5 µL), then incubated in the dark at room temperature for 15 min. Apoptosis rates were assessed by FCM within 1 h using a CytoFLEX flow cytometer (Beckman Coulter, USA) and data were analyzed with CytExpert software.

### JC‐1 Mitochondrial Membrane Potential

The cell apoptosis was detected using the JC‐1 Mitochondrial Membrane Potential Assay Kit (MCE, HY‐K0601) following the manufacturer's instructions. Cells were plated in 6‐well plates and cultured at 37 °C for 12 h, then the old medium was aspirated and replaced with 1 mL of fresh medium. 10 µL of JC‐1 (200 µM, equilibrated to room temperature before use) was added to each well, followed by gentle mixing and incubation at 37 °C for 20 min. Then the supernatant was removed, and the cells were washed twice with 1×PBS before resuspension in 500 µL of 1×PBS for fluorescence microscopy imaging.

### IP‐MS/MS

Mass spectrometry analysis was performed by SpecAlly Life Technology Co., Ltd (Wuhan, China) using a Thermo UltiMate 3000 RSLCnano coupled with a Q Exactive HF mass spectrometer. 786‐O cell lysates were split into two portions: one was immunoprecipitated with BCKDK, and the other with control IgG. IP efficiency was confirmed by Western blot. Samples with successful IP were subjected to mass spectrometry. The general protocol involved adding the reaction solution (1% SDC/100 mM Tris‐HCl, pH 8.5/10 mM TCEP/40 mM CAA) to the sample, incubating at 95 °C for 10 min to denature, reduce, and alkylate proteins. After centrifugation, the supernatant was diluted with an equal volume of ddH_2_O, and trypsin was added at a 1:50 mass ratio. Enzymatic digestion was carried out at 37 °C overnight, and digestion was terminated by adding TFA. After centrifugation, the supernatant was desalted and prepared for mass spectrometry analysis.

### Cytochrome C Release

Cytochrome c release was detected using the Cytochrome c releasing apoptosis assay kit (Abcam, ab65311). Cells from each group were collected, centrifuged, and washed with 1×PBS before being resuspended in cytosol extraction buffer. Then the cell suspension was homogenized and centrifuged at 750×g for 10 min. The supernatant was collected and centrifuged at 11000×g for 30 min to obtain the cytosolic fraction. The remaining pellet was resuspended in mitochondrial extraction buffer to isolate the mitochondrial fraction. Finally, both cytosolic and mitochondrial fractions were measured by Western blot.

### Immunohistochemistry (IHC)

The TMA of human RCC was purchased from Shanghai Outdo Biotech (Shanghai, China; HKidE150CS03, XT19‐021). The study protocol for human tissue research was reviewed and approved by the Medical Ethics Committee of the Seventh Affiliated Hospital of Sun Yat‐sen University (Approval No. KY‐2024‐314‐01). IHC staining of mouse tumor tissues was performed using BCKDK (1:50), Ki‐67 (1:1000), p‐AKT (Thr308) (1:50), p‐AKT (Ser473) (1:100), p‐mTOR (Ser2448) (1:100), cleaved caspase 3 (1:40), and caspase 3 (1:100) antibodies.

### RNA‐seq Analysis

RNA sequencing was performed by Gene Denovo Biotechnology Co., Ltd. (Guangzhou, China) using the Illumina NovaSeq 6000 platform. Total RNA was extracted from human BCKDK control and knockdown 786‐O cells and subsequently subjected to RNA sequencing. Standard sample handling procedures were followed as previously described.^[^ ^66^ ^]^

### In Vivo Tumor Xenograft

All animal studies adhered to the institutional animal care regulations of TopBiotech Co., Ltd. (Shenzhen, China) and complied with IACUC and AAALAC guidelines. The protocols were approved by the animal ethics committee of TopBiotech Co., Ltd. (Approval Nos. TOP‐IACUC‐2023‐0261 and TOPGM‐IACUC‐2024‐0102). BALB/c nude mice (4‐5 weeks, female) were obtained from Vitonglihua Experimental Animal Technology Co., Ltd. (Beijing, China) for in vivo xenograft studies. A total of 6 × 10^6^ shCtrl or shBCKDK#2 786‐O cells in 100 µL PBS mixed with Matrigel (1:1) were subcutaneously injected into the right flanks of the mice. For the xenograft tumor drug treatment studies, 5 × 10^6^ 786‐O cells in 100 µL PBS and Matrigel (1:1) were injected subcutaneously into the axillary region of the mice. When the average tumor volume reached 100 mm^3^, the mice were randomly assigned to four treatment groups: vehicle (PBS), BT2 (20 mg kg^−1^), DOX (8 mg kg^−1^), and the combination (BT2+DOX), with intraperitoneal injections every other day for 3 consecutive weeks. Tumor length was measured every 3 days, with the minimum (a) and maximum (b) dimensions used to calculate tumor volume using the formula: 0.5 × a^2^ × b. Mice were euthanized when the tumor volume reached 1000 mm^3^.

### Statistical Analysis

All in vitro data were derived from three independent experiments and are presented as mean ± standard deviation (SD). All Western blots were presented as mean ± SEM and representative pictures of three independent experiments. Statistical analyses were performed using an unpaired Student's *t*‐test or one‐way ANOVA, followed by Tukey's test. Statistical analysis was conducted using GraphPad Prism Software (version 9.0.2, USA). DFS curves for BCKDK expression in patients with KIRC were generated from the Kaplan–Meier plotter online database. A *p*‐value >0.05 was considered non‐significant (ns), while *p*‐values< 0.05, < 0.01, and < 0.001 were considered significant and denoted by^*^, ^**^, and ^***^, respectively.

## Conflict of Interest

The authors declare no conflict of interest.

## Author Contributions

Q.T., J.W., and Q.L. contributed equally to this work. Q.T. was responsible for the original draft writing, project administration, review and editing of the manuscript, visualization, formal analysis, data curation, and conceptualization. J.W. and Q.L. contributed to the review and editing of the manuscript, investigation, funding acquisition, data curation, visualization, and conceptualization. Y.C., Z.‐N.L., H.P., and C.‐Y.C. contributed to formal analysis, visualization, and data curation. Y.X., Y.L., J.F., and C.Q. were responsible for software development and contributed to conceptualization. L.F. and Z.X.X. contributed to visualization and data curation. Z.‐S.C. and Y.L. were responsible for validation, investigation, manuscript review and editing, methodology, formal analysis, and conceptualization. S.F., T.L., L.Z., and Y.P. contributed to supervision, funding acquisition, project administration, resources, investigation, and conceptualization.

## Supporting information



Supporting Information

Supporting Information

## Data Availability

The data that support the findings of this study are available on request from the corresponding author. The data are not publicly available due to privacy or ethical restrictions.
