# Supplementary material for: Branched‐Chain α Keto‐Acid Dehydrogenase Kinase‐Mediated AKT Phosphorylation Promotes RCC Tumorigenesis and Drug Resistance
Source: Adv Sci (Weinh). 2025 Aug 11;12(40):e11081. doi: 10.1002/advs.202411081 (PMC12561327; doi:10.1002/advs.202411081)
Supplement: Supplementary file 1 — Supporting Information [file ADVS-12-e11081-s001.docx]

**Branched-chain α keto-acid dehydrogenase kinase-mediated AKT phosphorylation promotes RCC** **tumorigenesis and drug resistance**

**Supplementary data**

**
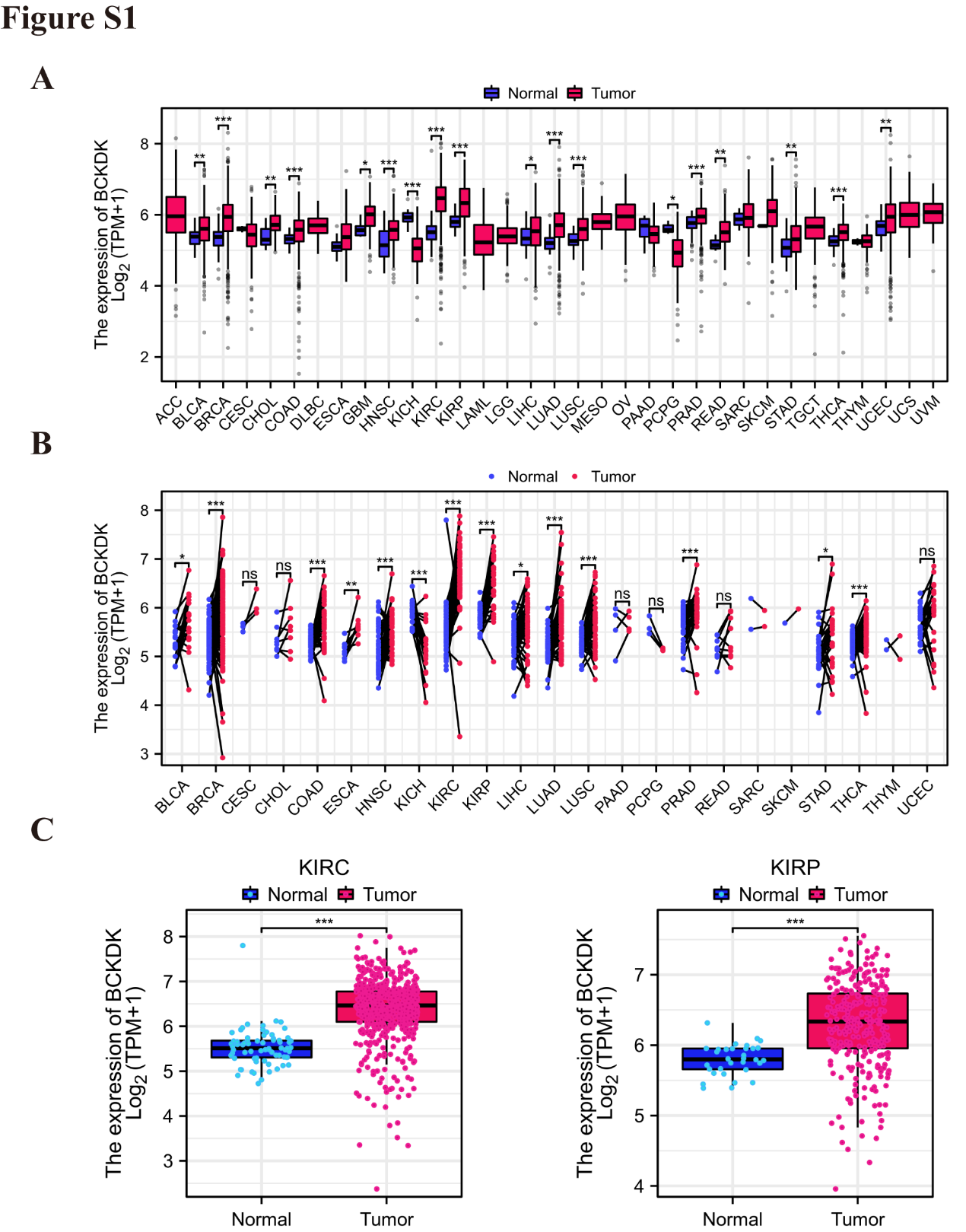
**

**Figure S1. BCKDK mRNA expression is upregulated in RCC. A.** BCKDK mRNA expression in pan cancer was analyzed by TCGA database. **B.** BCKDK mRNA expression in paired pan-carcinoma samples. **C**. The expression of BCKDK mRNA in tumor tissues was upregulated than that corresponding normal tissues in KIRC and KIRP.


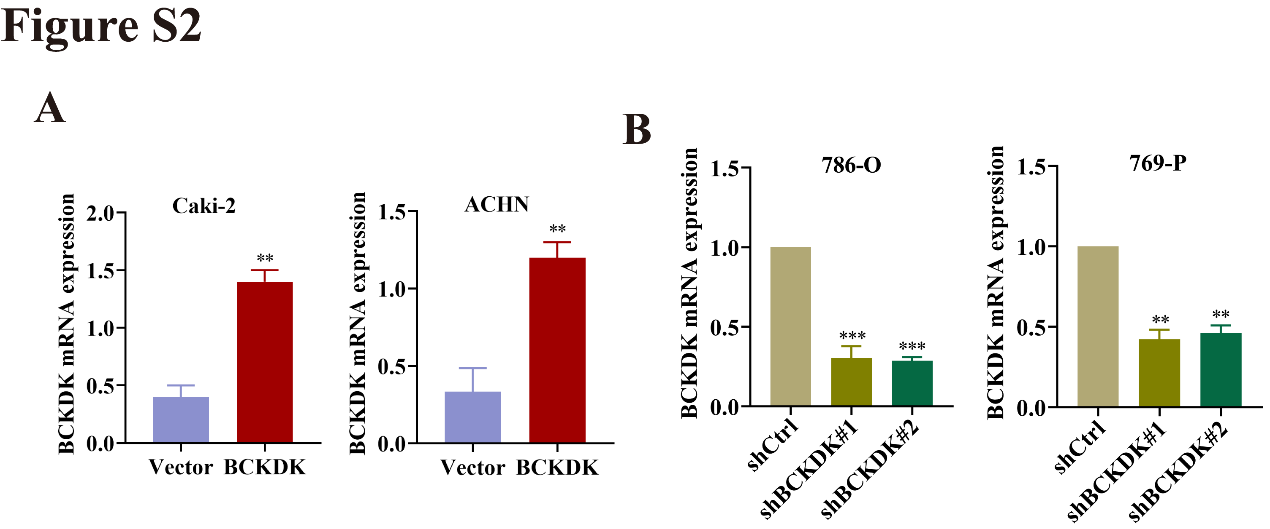


**Figure S2. RT-qPCR of BCKDK** **overexpression and knockdown RCC cell lines. A.** RT-qPCR analysis of BCKDK expression level indicated in Caki-2 and ACHN cell lines with stable BCKDK overexpression. **B.** RT-qPCR analysis of BCKDK expression indicated in 786-O and 769-P cell lines with BCKDK knockdown. *p* < 0.05, or < 0.01, or < 0.001 are regarded to be significant and marked with *, ** and *** respectively.


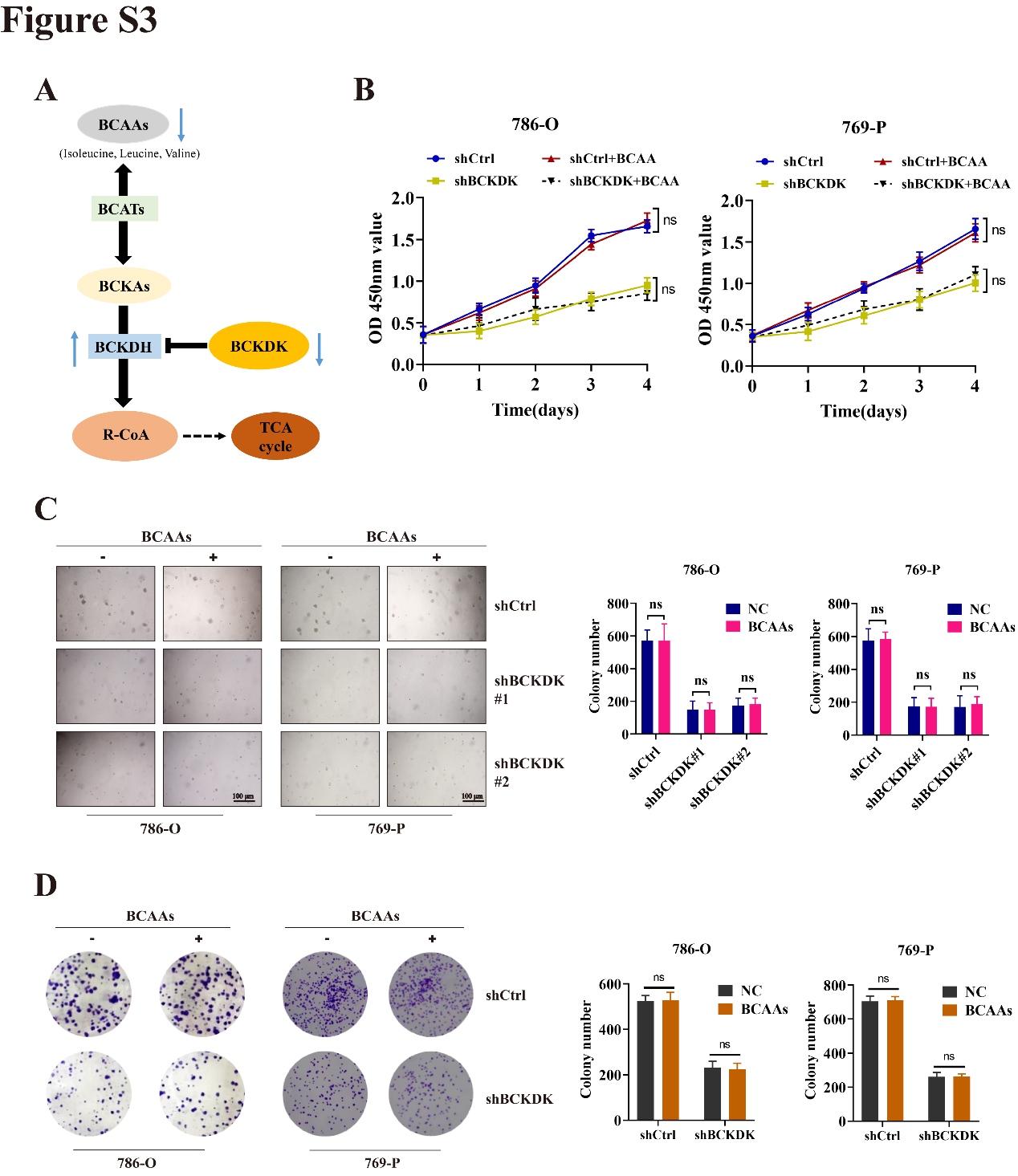


**Figure S3. BCAAs supplementation could not induce proliferation of RCC cells. A.** Pathway illustrating BCAAs (leucine, isoleucine, and valine) catabolism and the negative regulation of BCAAs breakdown by BCKDK kinase. BCAA transaminases 1and 2 (BCAT1/2) transfer nitrogen to α-ketoglutarate (α-KG) to produce glutamine and the specified branched-chain keto acid (BCKAs). These keto acids are next metabolized by BCKDH complex to produce branched-chain acyl-CoA (R-CoA) that can be further metabolized in several steps to the TCA cycle intermediates acetyl-CoA and/or succinyl-CoA. The enzyme activity of BCKDH is negatively regulated by BCKDK. **B.** Cell proliferation after supplied with BCAAs (leucine, isoleucine, and valine: 1: 1: 1) in BCKDK knockdown 768-O and 769-P cells were determined by CCK-8 assay. **C.** The effect of BCAAs supplementation on the colony formation of BCKDK knockdown 786-O and 769-P cells were analyzed by softagar assay. Scale bar = 100 µm. **D.** The influence of BCAAs supplementation on cell proliferation ability of BCKDK knockdown cells were investigated by plate colony formation. NC indicates negative control. NS indicates not significant.

**
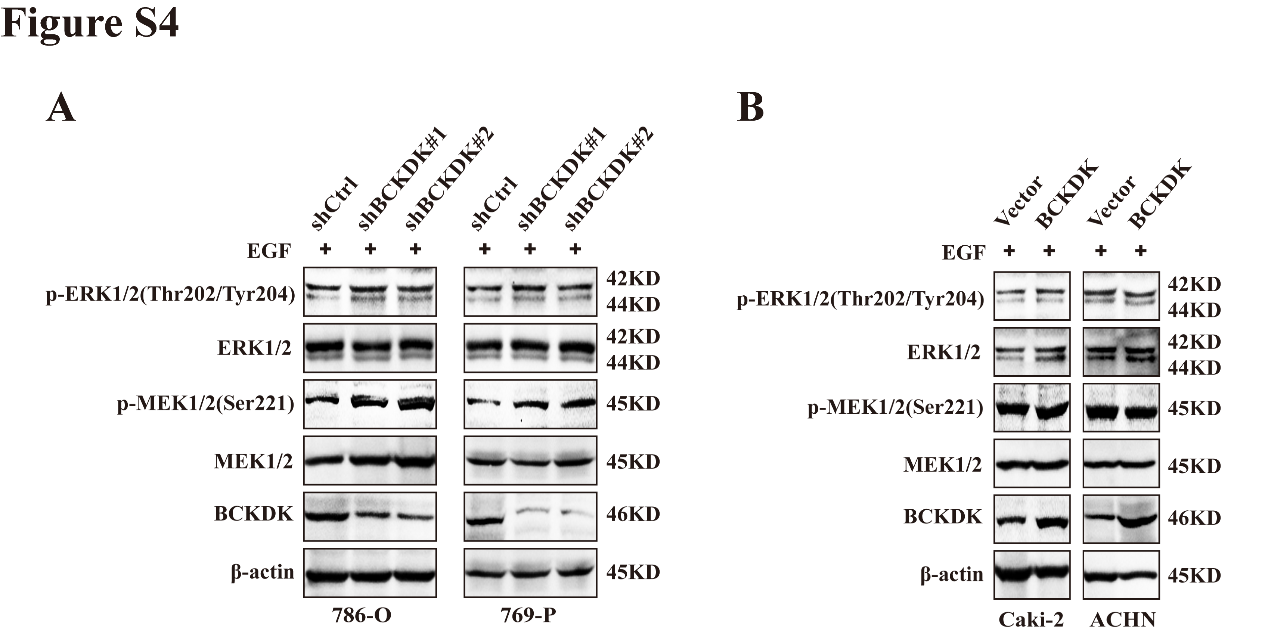
**

**Figure S4. BCKDK couldn’t activate the MEK/ERK signaling pathway in RCC cells.**

**A.** The expression level of p-MEK and p-ERK in 786-O and 769-P BCKDK knockdown cells was analyzed by Western blot. EGF: 15 min, 20 ng/mL. **B.** The expression level of p-MEK and p-ERK in Caki-2 and ACHN BCKDK overexpression cells was analyzed by Western blot. EGF: 15 min, 20 ng/mL.

**
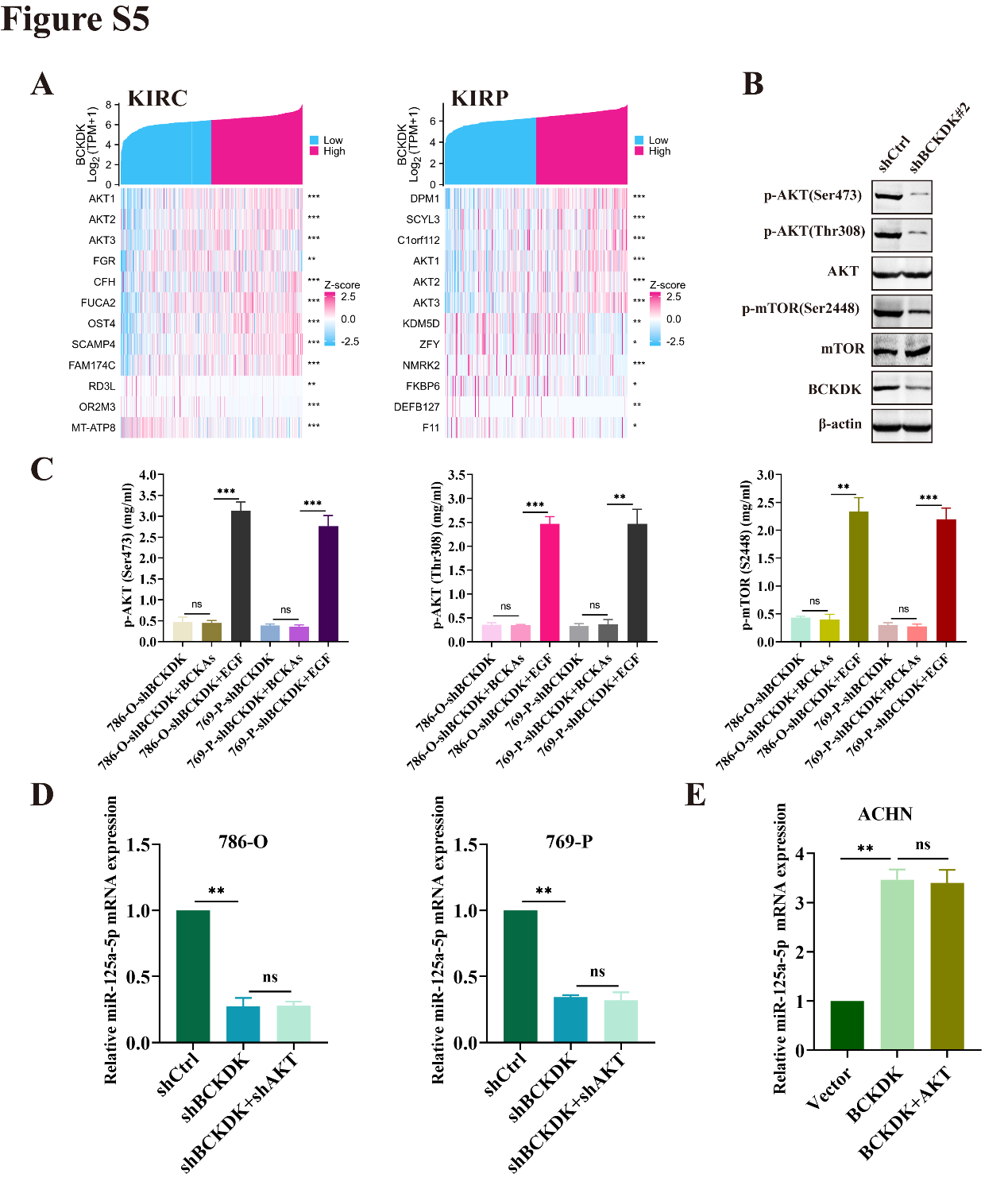
**

**Figure S5. BCKDK deficiency inhibits AKT/mTOR signaling pathway in vivo. A.** Correlation analysis on the co-expression of differentially expressed genes of BCKDK in KIRP and KIRC of TCGA database. **B.** The mouse subcutaneous tumor tissues were performed western blot to assess whether BCKDK silence inhibited the activation of the AKT/mTOR pathway in vivo. **C.** Quantification of p-AKT (Ser473), p-AKT(Thr308), and p-mTOR (Ser2448) in RCC cell lysates by sandwich ELISA. **D.** RT-qPCR analysis of miR-125a-5p expression indicated in 786-O and 769-P cell lines with BCKDK and AKT knockdown. **E.** RT-qPCR analysis of miR-125a-5p expression in in ACHN cell lines with stable BCKDK and AKT overexpression. *p* < 0.05, or < 0.01, or < 0.001 are regarded to be significant and marked with *, ** and *** respectively. NS indicates not significant.


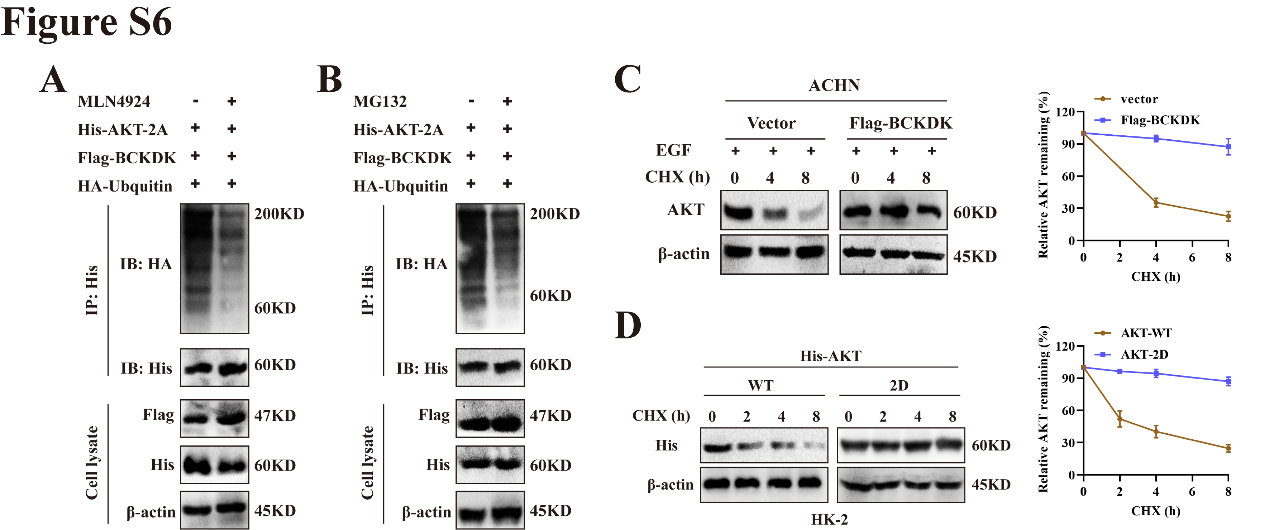


**Figure S6. Phosphorylation of AKT inhibits its ubiquitination-mediated degradation.** **A.** The ubiquitin-mediated degradation of AKT-2A were analyzed in HEK293T cells transfected with indicated plasmids followed by treated with or without MLN4924 (E3 ubiquitin ligase inhibitor, 1μM for 12 h). **B.** The ubiquitin-mediated degradation of AKT-2A were analyzed in HEK293T cells transfected with indicated plasmids followed by treated with or without MG132 (proteasome inhibitor, 10μM for 8 h). **C. D.** AKT protein stability assay in BCKDK-overexpressing ACHN and HK-2 cells treated with cycloheximide (CHX, 50μg/mL) for indicated time. Left: Representative blot; Right: Quantified levels normalized to β-actin.


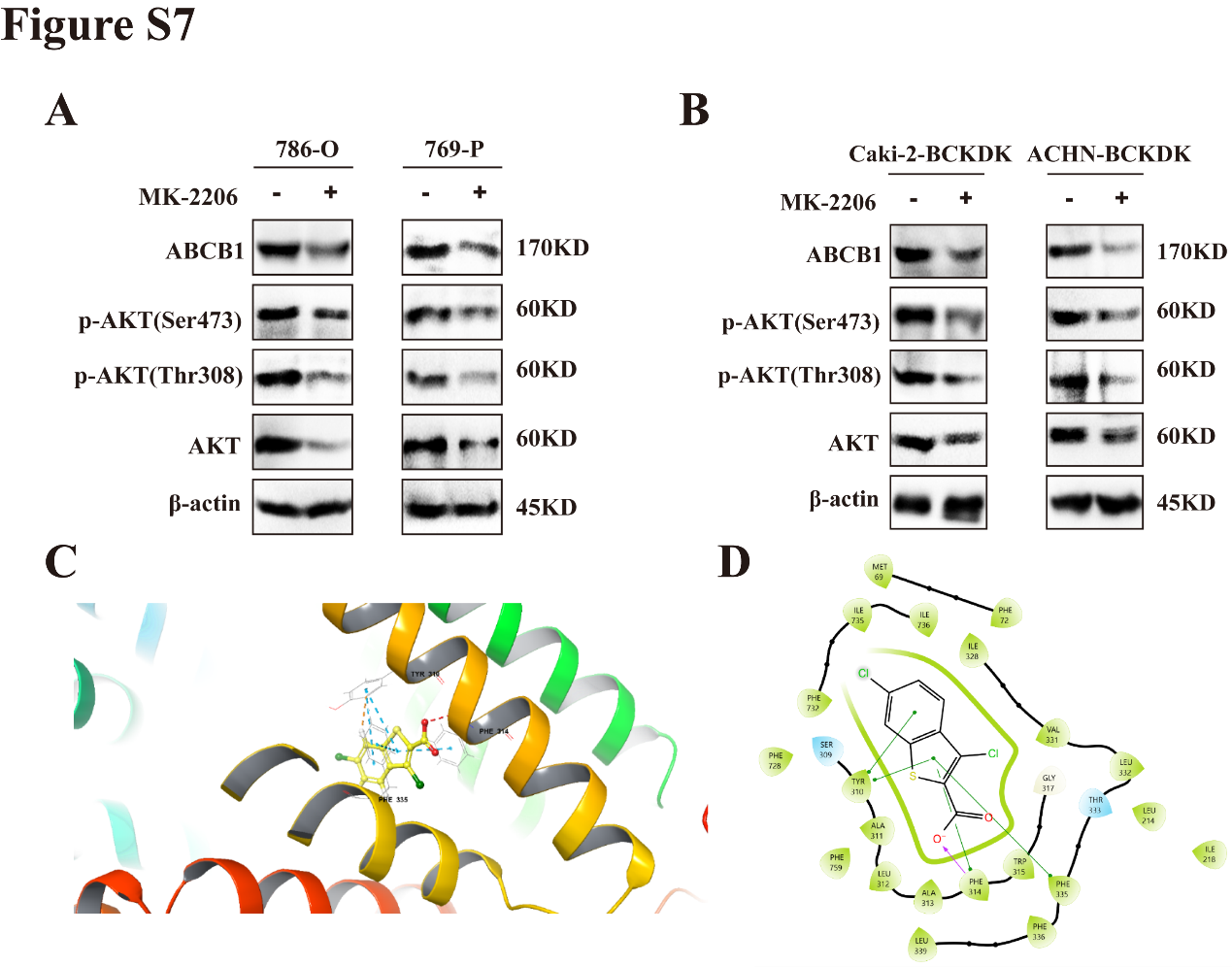


**Figure S7. BT2 exhibited high docking score towards ABCB1. A.** The expression level of ABCB1 in 786-O and 769-P cells was analyzed by Western blot. MK-2206: 6μM, 12h. **B.** The expression level of ABCB1 in Caki-2 and ACHN BCKDK overexpression cells was measured. MK-2206: 6μM, 24h. **C.** Docked position of BT2 with human ABCB1 transporter protein. **D.** Docked position of BT2 with human ABCB1 protein in two dimensions. The interaction was indicated as follows: π-π stacking interaction - the green line, hydrogen bond - purple arrow.


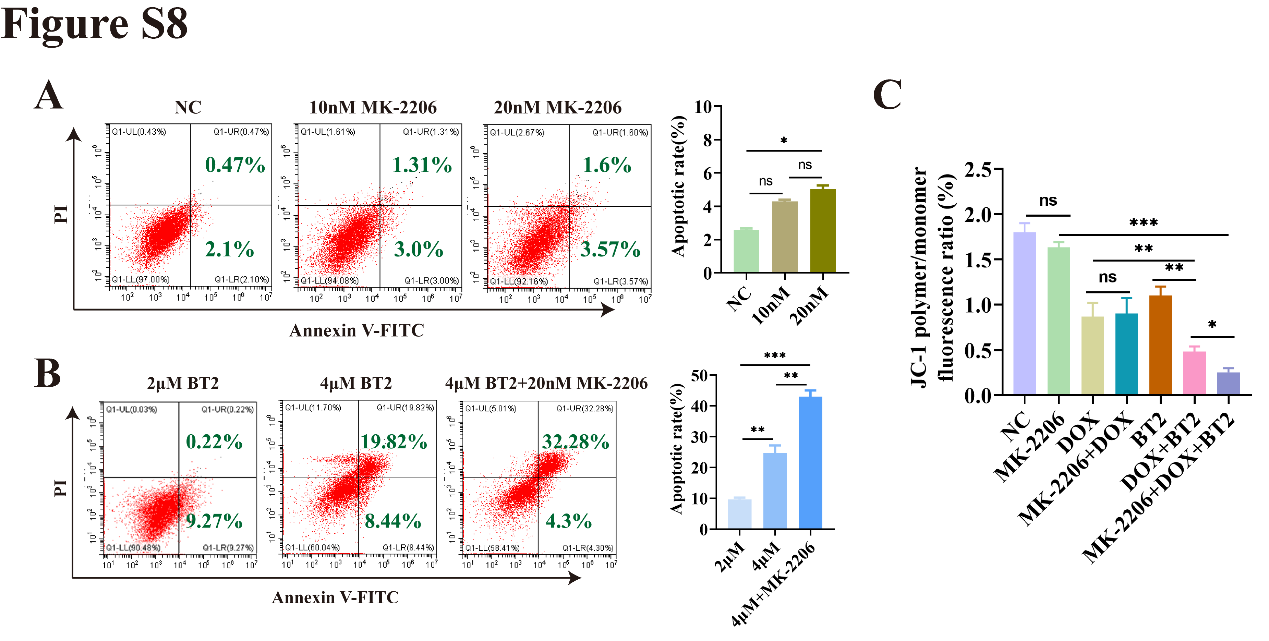


**Figure S8. Targeting AKT enhances BCKDK-mediated apoptosis in RCC cells. A.** Flow cytometry of RCC cells treated with MK-2206 at different concentrations (0, 10, and 20 nM). **B.** Apoptosis of RCC cells treated with BT2 and MK-2206 was detected by flow cytometry. The corresponding bar graphs represent statistical results from three independent replicate experiments, and the quantified data include both Annexin V and PI double-positive cells (late apoptotic cells) and Annexin V single-positive cells (early apoptotic cells). **C.** The mitochondrial membrane potential assay kit with JC-1 was used to assess changes in mitochondrial membrane potential in 786-O cells treated with MK-2206, BT2, and DOX alone or in combination. *p* < 0.05, or < 0.01, or < 0.001 are regarded to be significant and marked with *, ** and *** respectively. NS indicates not significant.

**
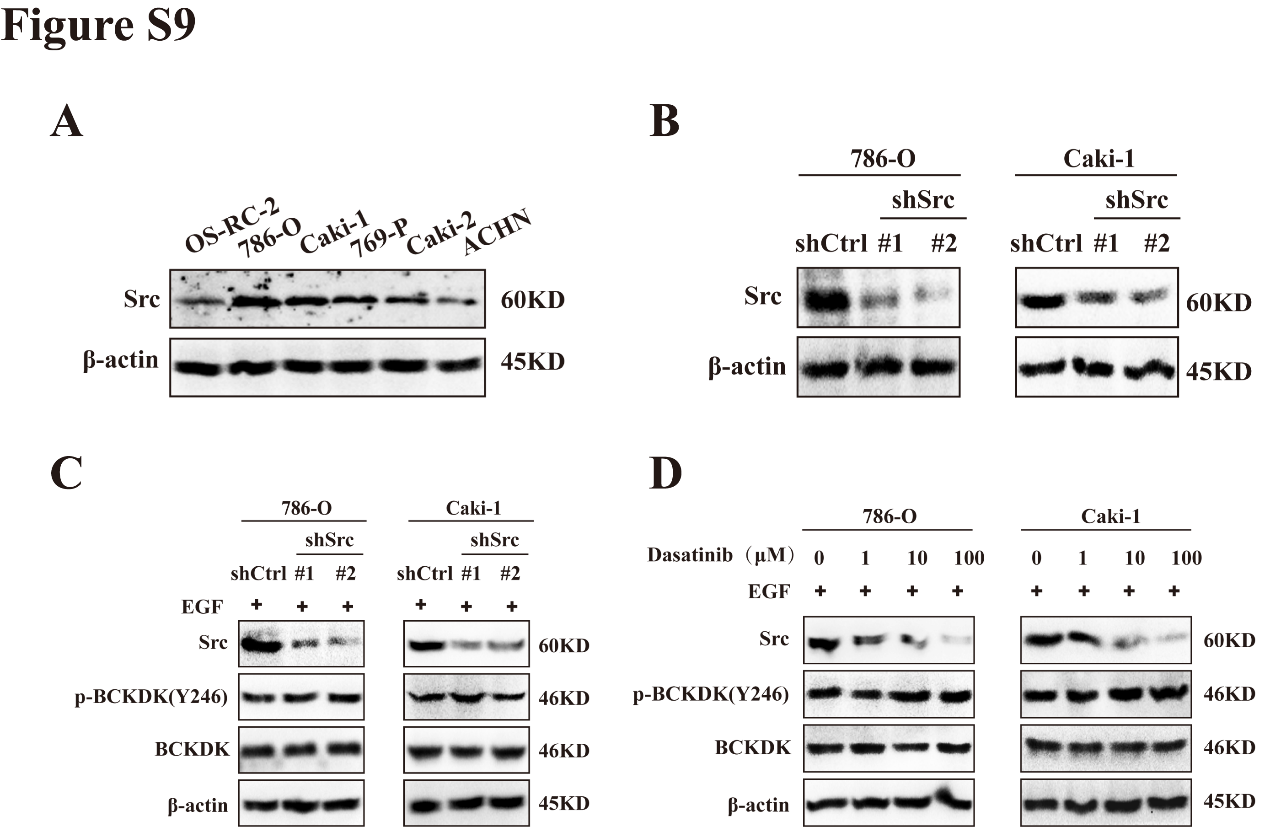
**

**Figure S9. Src deficiency did not affect BCKDK activity of RCC cells. A.** Expression of Src in six different RCC cell lines was detected by Western blot. **B.** Knockdown efficiency of Src in 786-O and Caki-1 cells was measured by Western blot. **C.** The effect of Src silence on BCKDK activation in RCC cells was accessed. **D.** 786-O and Caki-1 cells were treated with Dasatinib (Src inhibitor) at diverse concentrations (0, 1, 10, and 100µM), and the effect of Src-mediated BCKDK activation was analyzed by Western blot.
